# Utility of risk prediction models to detect atrial fibrillation in screened participants

**DOI:** 10.1093/eurjpc/zwaa082

**Published:** 2020-11-29

**Authors:** Michiel H F Poorthuis, Nicholas R Jones, Paul Sherliker, Rachel Clack, Gert J de Borst, Robert Clarke, Sarah Lewington, Alison Halliday, Richard Bulbulia

**Affiliations:** 1 Clinical Trial Service Unit and Epidemiological Studies Unit, Nuffield Department of Population Health, University of Oxford, Richard Doll Building, Old Road Campus, Oxford OX3 7LF, UK; 2 MRC Population Health Research Unit, Nuffield Department of Population Health, University of Oxford, Oxford, Old Road Campus, Oxford, OX3 7LF, UK; 3 Department of Vascular Surgery, University Medical Center Utrecht, Utrecht University, Heidelberglaan 100, 3584 CX, Utrecht, The Netherlands; 4 Nuffield Department of Primary Care Health Sciences, University of Oxford, Radcliffe Primary Care Building, Radcliffe Observatory Quarter, Woodstock Rd, Oxford OX2 6GG, UK; 5 Nuffield Department of Surgical Sciences, John Radcliffe Hospital, Headley Way, Oxford OX3 9DU, UK

**Keywords:** Atrial fibrillation, Risk prediction models, Stroke, Selective screening, External validation

## Abstract

**Aims:**

Atrial fibrillation (AF) is associated with higher risk of stroke. While the prevalence of AF is low in the general population, risk prediction models might identify individuals for selective screening of AF. We aimed to systematically identify and compare the utility of established models to predict prevalent AF.

**Methods and results:**

Systematic search of PubMed and EMBASE for risk prediction models for AF. We adapted established risk prediction models and assessed their predictive performance using data from 2.5M individuals who attended vascular screening clinics in the USA and the UK and in the subset of 1.2M individuals with CHA_2_DS_2_-VASc ≥2. We assessed discrimination using area under the receiver operating characteristic (AUROC) curves and agreement between observed and predicted cases using calibration plots. After screening 6959 studies, 14 risk prediction models were identified. In our cohort, 10 464 (0.41%) participants had AF. For discrimination, six prediction model had AUROC curves of 0.70 or above in all individuals and those with CHA_2_DS_2_-VASc ≥2. In these models, calibration plots showed very good concordance between predicted and observed risks of AF. The two models with the highest observed prevalence in the highest decile of predicted risk, CHARGE-AF and MHS, showed an observed prevalence of AF of 1.6% with a number needed to screen of 63. Selective screening of the 10% highest risk identified 39% of cases with AF.

**Conclusion:**

Prediction models can reliably identify individuals at high risk of AF. The best performing models showed an almost fourfold higher prevalence of AF by selective screening of individuals in the highest decile of risk compared with systematic screening of all cases.

**Registration:**

This systematic review was registered (PROSPERO CRD42019123847).

## Introduction

Atrial fibrillation (AF) is the most frequent sustained cardiac arrhythmia in clinical practice and its prevalence is increasing, due to ageing populations, altered lifestyle habits and increasing levels of adiposity. Over 33.5 million people worldwide are currently diagnosed with AF.^1^ AF may be categorized in different ways, including by the frequency of the arrhythmia as either paroxysmal, persistent, or permanent. However, all subtypes are associated with an increased risk of stroke and other cardiovascular disease outcomes, which include a five-fold higher risk of cardioembolic stroke.^2^^,^^3^

Risk prediction scores such as CHA_2_DS_2_-VASc are recommended to help determine the stroke risk for people who are diagnosed with AF, categorized as low, medium, or high.^4^ Anticoagulation with either a vitamin K antagonist such as warfarin or a direct oral anticoagulant in high-risk individuals can reduce their stroke risk by around 65%.^5^ Yet many people with AF currently go undetected, either because they are asymptomatic or have paroxysmal disease not detected at the time of assessment. A recent systematic review of single time-point screening reported a prevalence of undetected AF of 1.4% in adults aged ≥65 years old in the general population.^6^ However, AF is typically found in up to 20% of cases with ischaemic stroke.^7^^,^^8^ In at least half of such cases, AF is newly diagnosed at the time of the event.^9^^,^^10^ This has prompted interest in implementing national screening programmes to detect people with AF, particularly in individuals who might benefit from anticoagulation.^4^^,^^11^^,^^12^

One argument against population-level systematic screening is the low overall prevalence of AF in the general population. Accurate identification of individuals at higher risk of AF could help to target screening, reduce the number needed to screen. Most simply, this involves screening above a certain age threshold given the increased prevalence of AF in older people; over 80% of cases with AF occur in individuals aged over 65 years compared to 2.8% who are aged below 45 years.^13^ Currently, international guidelines suggest either opportunistic screening in individuals aged 65 years or older, or systematic screening in those aged 75 years or older and individuals at high-risk of stroke since the latter approach has been shown to be particularly cost-effective.^14–16^

Risk prediction models have been developed to detect either incident or prevalent AF and may be able to more accurately identify populations at high risk of AF to inform selective screening. These have the additional benefit of identifying people who are also at higher risk of stroke and therefore likely to benefit from treatment.^17^^,^^18^ Assessing the predictive performance of such models is necessary before seeking to implement these approaches to determine their comparative accuracy and utility. We conducted a systematic review of established risk prediction models of AF and then evaluated the predictive performance of these models in a large contemporary screened population.

## Methods

We conducted a systematic review according to a predefined protocol to identify established prediction model to detect AF. This protocol has been registered prospectively in the international prospective registry for systematic reviews (PROSPERO): CRD42019123847. We report the results of our systematic review consistent with the Preferred Reporting Items for Systematic Reviews and Meta-analysis (PRISMA).^19^

### Search strategy and eligibility criteria

We searched Medline (via PubMed interface) and EMBASE (via OVID interface) from inception to 1 March 2019 using comprehensive electronic strategies, which incorporated a validated search filter (Supplementary material online, *eTable 1*). We included articles that: (i) develop risk prediction models for the prevalence or incidence of AF based on multiple risk factors; (ii) used general or screened population as domain, not diseased populations at higher risk of AF; (iii) used a single time-point 12-lead electrocardiogram (ECG) for diagnosing AF; and (iv) published in peer-reviewed journals without any language restrictions.

### Screening process and data extraction

Two authors (M.H.F.P. and N.R.J.) independently screened all titles and abstract of the retrieved references and subsequently independently reviewed full-texts for final inclusion in this study. Discrepancies could be resolved in those meetings with the help of a third author (R.B.) where required. We performed backward citation searching using the bibliographies of included studies.

Two authors (M.H.F.P. and N.R.J.) independently extracted the following data from the included studies that report the development of a risk prediction model, based on the CHARMS checklist:^20^ source of data, setting study, geographic area (country and continent), study years, sample size, modelling method (e.g., logistic model), number of participants with missing data, handling of missing data, investigation of satisfaction of modelling assumptions, selection methods for predictor selection, shrinkage of predictor weights, number of outcome events, number of patients, ascertainment of outcome, number and type of predictors used in the final model, number of outcome events per variable, presentation of model, model performance (calibration and validation).

### Validation cohort

A cohort of self-referred and self-funded individuals who attended commercial vascular screening clinics (Life Line Screening Inc.) between 2008 and 2013 in the USA and UK was used to assess the predictive performance. All individuals completed standardized questionnaires including questions about their age, sex, smoking status, alcohol use, height and weight, history of vascular disease (coronary artery disease, congestive heart failure, stroke, transient ischaemic attack, and peripheral arterial disease), valvular disease, chronic obstructive pulmonary disease, hypertension and use of antihypertensive medication, and diabetes mellitus. Blood pressure was measured as part of the ankle-brachial pressure index assessment. Standard blood pressure cuffs and sphygmomanometers were used, systolic blood pressure (SBP) being measured using a Doppler probe.

### Predicted outcome and its ascertainment

The predicted outcome was the prevalence of AF, measured with a single 12-lead ECG. All ECGs were evaluated by physicians who received in-house training.

### Statistical analyses (external validation)

Characteristics of the predictor variables in the included models were summarized using standard methods. We excluded participants with an established history of AF prior to screening (*N* = 285 934), who did not undergo a single 12-lead ECG (*N* = 356 684), or with inconsistent values for sex (*N* = 14 287). We used the same population for all analyses to enable comparisons between different models. Some models applied age and body mass index (BMI) restrictions (Supplementary material online, *eTable 2*). We therefore further excluded participants who were younger than 45 at screening (*N* = 59 357) or who had a BMI lower than 18 (*N* = 18 175).

Variables only relevant for predicting incident AF, such as ECG and echocardiographic characteristics, were not included in our assessment of the risk prediction models. Predictors involving biochemical or other blood measurements were not included, since their availability for inclusion in screening programmes or measurement before performing a single ECG might limit the clinical applicability (Supplementary material online, *eTable 3*). We used proxies whenever possible and appropriate for any predictors that were not available in our dataset. Predictors for which no proxy was found were considered missing (Supplementary material online, *eTable 3*).

Missing data were imputed if data were missing in <30% (Supplementary material online, *eTable 4*). We used chained equations and created 20 imputed datasets with 200 iterations.^21^ BMI was calculated before imputation.^22^ Post-imputation rounding was applied to limited-range variables (SBP, heart rate, BMI, height, and weight), if needed.^23^ Analyses were performed in the resulting 20 imputed datasets.

We used the risk equations to calculate the probability of AF for each participant. We used the β-coefficients (predictor weights) of prediction models that were based on logistic regression or time-dependent regression modelling, such as cox regression (Supplementary material online, *eTable 5*). We also calculated a sum score (total points) for each participant by summing the points assigned to each predictor of the score chart.

We examined the discrimination and calibration indices of the prediction models, assessed using the area under the receiver operating characteristic (AUROC) curve and calibration plots respectively. We calculated the AUROC curve per imputed dataset and results were pooled using Rubin’s rules.^24^^,^^25^ For models that reported the risk equation, we estimated the mean probability per participant across the 20 imputed datasets and subsequently we split the predicted risks in deciles and calculated observed probability with corresponding 95% confidence interval (CI) per decile. We recalibrated the prediction models to the prevalence of AF in our cohort by re-estimating the intercept. This type of recalibration is referred to as ‘update intercept’ or ‘calibration-in-the-large’.^26^ For this, we fitted a logistic model with a fixed calibration slope and the intercept as the only free parameter.

In addition, for models that reported a score chart, we created bar charts with the observed prevalence of AF by sum score.

We performed additional assessments of discrimination and calibration using participants with CHA_2_DS_2_-VASc of two or more, since anticoagulation is recommended for these people if AF is found.^14^

### Test characteristics and reclassification measures

We assessed two possible cut-offs for a selective screening. We assessed test characteristics, such as sensitivity, specificity, positive predictive value, negative predictive value, prevalence, and number needed to screen (NNS), of selective screening of the 10% and 20% individuals at highest predicted risk of AF.

We calculated reclassification measures to assess the ability of the included risk prediction models to correctly identify cases with and without AF compared to the threshold of ≥65 years of age.^27^ We calculated integrated discrimination improvement (IDI), relative IDI (rIDI), and continuous net reclassification improvement (NRI).^27^^,^^28^ IDI is the absolute difference in discrimination slopes of the risk prediction models and the age threshold. rIDI is the ratio of absolute difference in discrimination slopes of the risk prediction models and the age threshold over the discrimination slope of the age threshold. Continuous NRI is the sum of the net percentages of participants with and without the AF correctly assigned a different predicted risk with the risk prediction models compared to the age threshold. Positive values correspond to improved classification. The reclassification measures were estimated for all 1000 bootstrap replications in each imputed dataset and the median value across the combined 20 datasets is reported (with the 95% CI obtained from the 2.5th and 97.5th percentiles). *P*-values <0.05 were considered significant. STATA version 15.1 was used for all statistical analyses and R version 3.5.1 was used for constructing the figures.

### Sensitivity analyses

We performed additional assessment of the prediction models in complete cases.

## Results

**Figure 1:**
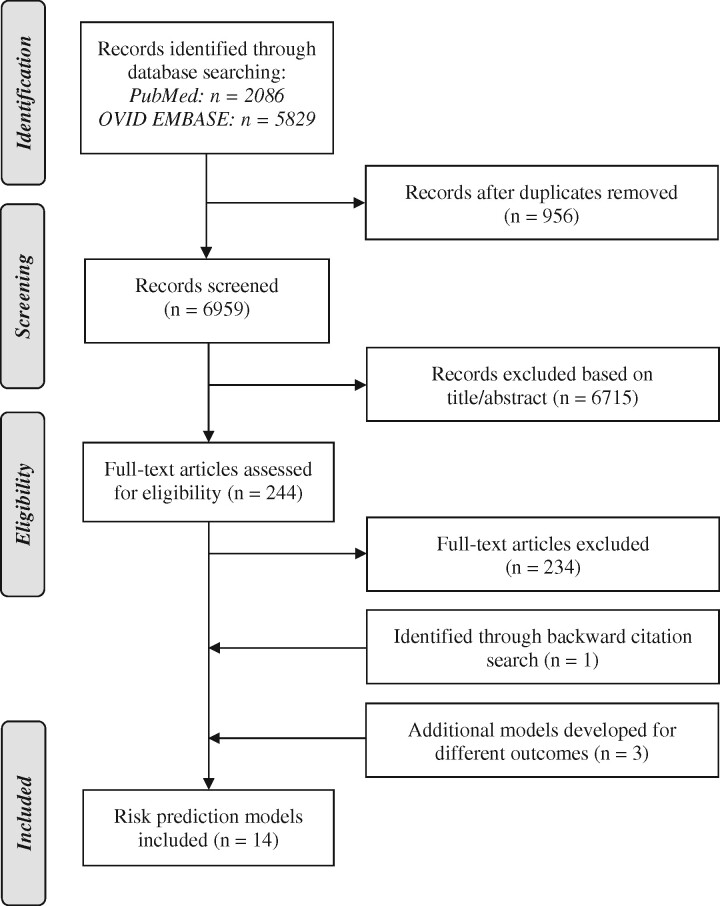
Flowchart.

We screened 6961 unique reports identified by our literature search, assessed 249 full-texts, and included 14 studies (*Figure 1* and Supplementary material online, *eTable 6*).^4^^,^^12^^,^^29–40^ Six studies used incident AF as predicted outcome,^32–37^ three used incident AF or atrial flutter,^29^^,^^30^^,^^39^ one used prevalent AF,^38^ and one did not specify the type of AF.^31^ HATCH was developed to predict progression to sustained AF and CHADS_2_ and CHA_2_DS_2_-VASc were developed to predict the risk of stroke in cases with AF.^4^^,^^12^^,^^40^ These three prediction models were included, although not originally designed for detecting AF, because they have been used in a number of subsequent studies for predicting AF and might be used for combined prediction of outcomes.^37^^,^^38^^,^^41^^,^^42^ Characteristics of model development are provided in *Table 1*.

**Table 1:** Selected characteristics of studies assessing different risk prediction models for AF

Author, year, and study name	Predicted outcome	Country	Cases/participants in derivation cohort (%)	Number of predictors^a^
Alonso *et al.*, 2013 (CHARGE-AF)^29^	Incident AF or atrial flutter	USA	1186/18 556 (6.39%)	11
Aronson *et al.*, 2018 (MHS)^30^	Incident AF or atrial flutter	Israel	5660/96 778 (5.8%)	10
Brunner *et al.*, 2014 (MAYO)^31^	AF	—	—	7
Chamberlain *et al.*, 2011 (ARIC)^32^	Incident AF	USA	515/14 546 (3.54%)	12
Ding *et al.*, 2017 (JINAN)^33^	Incident AF	China	134/33 186 (0.4%)	4
Everett *et al.*, 2013 (WHS)^34^	Incident AF	USA	404/13 743 (2.9%)	6
Hamada *et al.*, 2019 (SEIREI)^35^	Incident AF	Japan	349/65 984 (0.53%)	7
Kokubo *et al.*, 2017 (SUITA)^36^	Incident AF	Japan	311/6864 (4.5%)	9
Li *et al.*, 2018 (C_2_HEST)^37^	Incident AF	China	921/471 446 (0.20%)	6
Linker *et al.*, 2018 (SAAFE)^38^	Prevalent AF	USA	509/3790 (13.4%)	13
Schnabel *et al.*, 2009 (FHS)^39^	Incident AF or atrial flutter	USA	457/4764 (9.6%)	7
de Vos *et al.*, 2010 (HATCH)^40^	Progression to sustained AF	—	—	5
Gage *et al.*, 2001 (CHADS_2_)^12^	Stroke risk	—	—	5
Lip *et al.*, 2010 (CHA_2_DS_2_-VASc)^4^	Stroke risk	—	—	7

AF, atrial fibrillation.

a Number of predictors of the risk prediction models assessed in the present study are provided.

**Figure 2:**
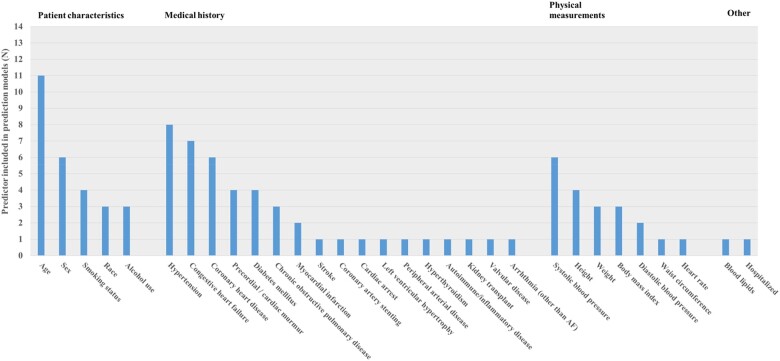
Included predictors. An overview of predictors used in the eleven risk prediction models that were developed to predict atrial fibrillation.

The number of predictors in the models varied from four to thirteen. An overview of predictors of the included prediction models originally developed for detecting AF is provided in *Figure 2*. Age was used as predictor in all of the models. Other predictors frequently included were hypertension (*n* = 8), heart failure (*n* = 7), coronary heart disease (*n* = 6), sex (*n* = 6), and SBP (*n* = 6). Of the fourteen included prediction models, predictor weights of 12 models were reported and score charts of eleven models.

### Validation cohort

The validation cohort consisted of 2 541 702 participants, of whom 10 464 (0.4%) had AF. In total, 1 153 878 (52.4%) participants had a CHA_2_DS_2_-VASc score of two or higher of which 5298 (0.5%) of the participants with AF. The mean CHA_2_DS_2_-VASc score was two in participants without AF and three in participants with AF. Characteristics of our cohort that were used as predictors in the included prediction models are provided in *Table 2*.

**Table 2:** Characteristics of variables used as predictors in the prediction cohort

	All participants (*N* = 2 541 702)	Participants with AF (*N* = 10 464)	Participants without AF (*N* = 2 531 238)
Age (years)	64.8 ± 9.6	72.9 ± 9.4	64.8 ± 9.6
Female sex	1 648 242 (64.8)	4315 (41.2)	1 643 927 (64.9)
Current smoker	219 444 (9.7)	751 (8.3)	218 693 (9.7)
Former smoker	693 974 (30.6)	3340 (36.7)	690 634 (30.5)
Never smoked	1 357 094 (59.8)	5012 (55.1)	1 352 082 (59.8)
Medical history			
Hypertension	1 015 663 (41.8)	5014 (51.9)	1 010 649 (41.8)
Antihypertensive medication	1 023 749 (43.4)	5317 (56.5)	1 018 432 (43.3)
DM	276 051 (11.9)	1622 (17.7)	274 429 (11.8)
CHD^a^	137 508 (6.2)	1156 (12.9)	136 352 (6.1)
Valvular disease	76 985 (4.0)	494 (6.9)	76 491 (4.0)
CHF	20 847 (0.9)	426 (4.8)	20 421 (0.9)
COPD	64 592 (3.4)	486 (6.8)	64 106 (3.4)
PAD	91 823 (3.7)	938 (9.5)	90 885 (3.6)
Stroke or TIA	78 048 (3.5)	819 (9.4)	77 229 (3.5)
Physical measurements			
Height (m)	1.7 ± 0.1	1.7 ± 0.1	1.7 ± 0.1
Weight (kg)	79.1 ± 18.2	86.5 ± 21.1	79.1 ± 18.2
BMI (kg/m^2^)	27.9 ± 5.3	28.9 ± 5.7	27.9 ± 5.3
SBP (mmHg)	133 ± 19.7	139 ± 21.2	133 ± 19.7
Heart rate (beats/min)	66 ± 10.3	77 ± 16.7	66 ± 10.3
CHA_2_DS_2_-VASc of ≥2	1 153 878 (52.4)	5298 (60.9)	1 148 580 (52.4)
Mean CHA_2_DS_2_-VASc	2 ± 1.3	3 ± 1.6	2 ± 1.3

Values are mean ± SD for continuous variables and *n* (%) for categorical variables.

AF, atrial fibrillation; BMI, body mass index; CHD, coronary heart disease; CHF, congestive heart failure; COPD, chronic obstructive pulmonary disease; DM, diabetes mellitus; PAD, peripheral arterial disease; SBP, systolic blood pressure; TIA, transient ischaemic attack.

a CHD is defined as previous myocardial infarction or a coronary intervention (bypass, angioplasty, or stenting).

### Predictive performance in validation cohort

#### Discrimination

**Figure 3:**
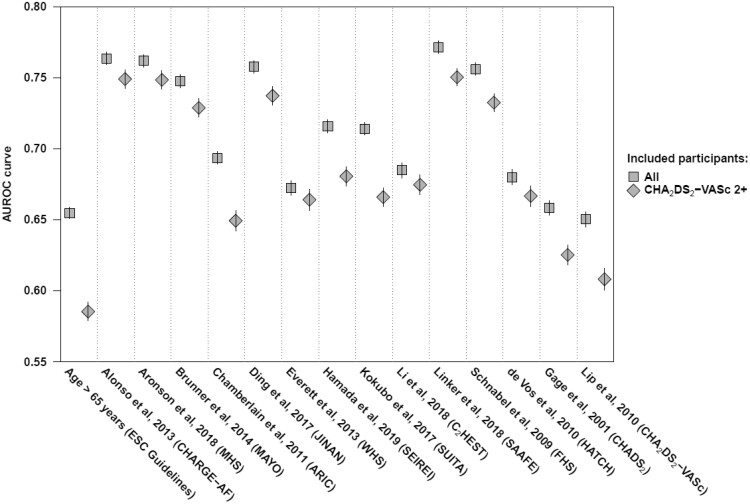
Discriminative performance. Squares represent the AUROC curves in the analysis of all 2.5M participants and diamonds in 1.2M participants with CHA_2_DS_2_-VASc of two or more.^4^ The vertical bars represent the 95% CIs. The AUROC curves are based on the regression equation in 12 prediction models,^29–40^ and on the point chart for two prediction models.^4^^,^^12^ Values are provided in Supplementary material online, *eTable 9*.

For discrimination in all participants, AUROC curves were between 0.71 and 0.77 in eight models,^29–31^^,^^33^^,^^35^^,^^36^^,^^38^^,^^39^ and between 0.65 and 0.69 in six models.^4^^,^^12^^,^^32^^,^^34^^,^^37^^,^^40^ (*Figure 3* and Supplementary material online, *eTable 7*). All models showed a statistically significant better discrimination compared with the age threshold of 65 years or older suggested for opportunistic screening in the current European Society of Cardiology (ESC) guidelines.^14^ All the models also had a statistically significant better discrimination than both CHADS_2_ and CHA_2_DS_2_-VASc.^4^^,^^12^

In participants with CHA_2_DS_2_-VASc scores of two or higher, AUROC curves were between 0.73 and 0.75 in six studies,^29–31^^,^^33^^,^^38^^,^^39^ and between 0.65 and 0.68 in six studies.^32^^,^^34–37^^,^^40^ The AUROC curve for the age threshold was 0.59 (95% CI 0.58-0.59).^14^ (*Figure 3* and Supplementary material online, *eTable 7*). The difference in discrimination between age alone and all other models was also statistically significant.

#### Calibration

**Figure 4:**
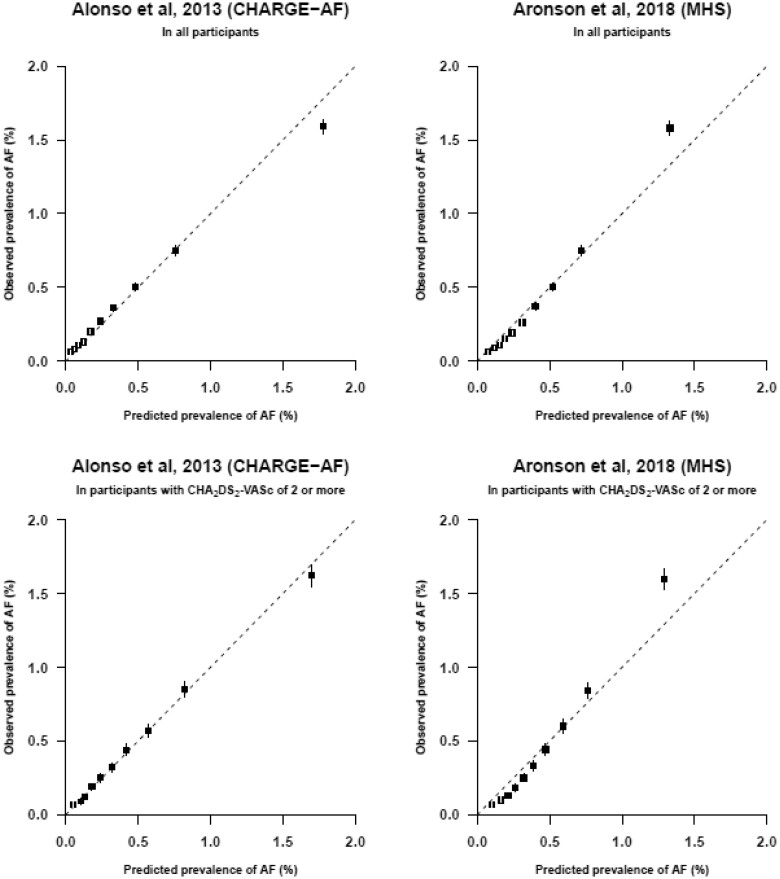
Calibration plots. Calibration plots of the two risk prediction models with the highest observed prevalence of AF in the highest decile of predicted risk: CHARGE-AF and MHS.^29^^,^^30^ To construct the calibration plots, data of all 2.5M participants (*top row*) and 1.2M participants with CHA_2_DS_2_-VASc of two or more (*bottom row*) were used. Mean predicted risk against the observed risk of AF across deciles of predicted risk (after recalibration with adjusting the intercept) is shown. The boxes represent the mean predicted risk for each decile and the vertical lines represent the 95% confidence intervals. The dotted diagonal line indicates perfect calibration. Boxes above the diagonal line indicate underestimation of risk and below the diagonal line overestimation of risk. The prevalences and number of cases of each decile are provided in Supplementary material online, *eTable 9*.

Calibration showed good correspondence between predicted and observed risks of AF in six of the eight models with AUROC curves >0.70.^29–31^^,^^33–36^^,^^39^ (*Figure 4* and Supplementary material online, *eFigure 1*). The two models with the highest observed prevalence in the highest decile of predicted risk were CHARGE-AF and MHS. An observed prevalence of AF of 1.6% was found in this decile (*Figure 4*).^29^^,^^30^ Prevalences were predicted accurately across all deciles of predicted risk except for the highest decile, where CHARGE-AF overestimated the observed prevalence (1.8% vs. 1.6%) and MHS underestimated the observed prevalence of AF (1.3% vs. 1.6%). In participants with CHA_2_DS_2_-VASc scores of two or higher, calibration plots showed similar results (*Figure 4*).

The predictors included in CHARGE-AF are age, ethnicity, height, weight, SBP, diastolic blood pressure, smoking, antihypertensive medication use, diabetes, heart failure and myocardial infarction, of which ethnicity and diastolic blood pressure were not included in the present analysis. The predictors included in MHS are age, sex, BMI, myocardial infarction, peripheral arterial disease, treated hypertension, SBP, chronic obstructive lung disease, female with autoimmune or inflammatory disease and heart failure by age group, of which female with autoimmune or inflammatory disease was not included in the present analysis. Other calibration plots are provided in Supplementary material online, *eFigure 1*. The bar charts showed increasing observed prevalence with increasing sum scores (Supplementary material online, *eFigure 2*).

### Test characteristics

We assessed selective screening of participants in the highest decile and highest two deciles of predicted risk. The prevalence of AF in the highest decile of predicted risk varied from 1.0% to 1.6% with corresponding NNS of 96 to 63 across the 12 prediction models (Supplementary material online, *eTable 10*). CHARGE-AF and MHS showed the highest observed prevalence of 1.6% by selective screening of these 10% highest risk cases. This identified 39% of cases with prevalent AF with a specificity of 90%.

**Figure 5:**
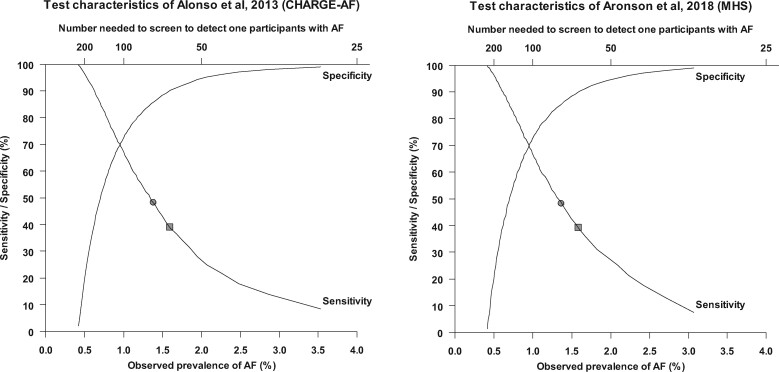
Test characteristics. Graph showing the sensitivity and specificity and corresponding observed prevalence and number needed to screen to detect 1 participant with AF using the prediction model developed by Alonso *et al*. 2013 (*left*) and Aronson *et al*. 2018 (*right*). The squares and circles correspond to selective screening of participants in the highest decile and highest two decile of predicted risk, respectively.

The prevalence of AF in the highest two deciles of predicted risk varied from 0.9% to 1.3% with corresponding NNS of 107 to 76 across the 12 prediction models. CHARGE-AF and MHS showed the highest observed prevalence of 1.3% by selective screening of these 20% highest risk cases. This identified 48% of cases with prevalent AF with a specificity of 85% (Supplementary material online, *eTable 10*). Observed prevalence, NNS, sensitivity and specificity for other cut-offs of predicted risk using CHARGE-AF and MHS are shown in *Figure 5*.

### Reclassification measures

Reclassification measures demonstrated a significant improvement of the CHARGE-AF and MHS prediction models compared to the age threshold of 65 years.^14^ For the CHARGE-AF risk prediction model, the IDI was 0.0048 (95% CI 0.0046–0.0051; *P* < 0.00001), rIDI was 1.84 corresponding to an 184% improved classification, and the NRI was 0.6201 (95% CI 0.6011–0.6387; *P* < 0.00001). For the MHS risk prediction model, the IDI was 0.0021 (95% CI 0.0020–0.0022; *P* < 0.00001), rIDI was 0.80 corresponding to an 80% improved classification, and the NRI was 0.4447 (0.4258–0.4643; *P* < 0.00001).

### Sensitivity analysis

Discrimination values were only marginally decreased in subsets with complete cases (Supplementary material online, *eTable 8*).

## Discussion

Our study is the first to compare the performance of all established risk prediction models for prevalent AF. We conducted an external validation in a large contemporary screened population who underwent a single time point 12-lead ECG to detect AF. Eight models showed AUROC curves of >0.70 and in seven of these, there was good concordance of predicted and observed risks. Several common predictors were included in most models, such as age, hypertension and heart failure. The two models with the highest observed prevalence of AF in the highest decile of predicted risk were developed in the CHARGE-AF and MHS cohorts.^29^^,^^30^ The observed prevalence of AF in the highest deciles across the two models was 1.6%, with a number needed to screen to detect one case with AF of 63. This was almost four-fold higher than the overall prevalence and 25-fold higher than the lowest decile of predicted risk. These prediction models showed better discriminative performance compared to an age threshold of 65 years, CHADS_2_ and CHA_2_DS_2_-VASc. Application of these risk models therefore may be able to inform more selective opportunistic or systematic screening.

Unselected population screening is likely to detect only small numbers of people with AF. For example, the recent Apple Heart Study screened nearly 420,000 people using smartwatch technology with an irregular pulse notification system.^43^ Possible cases wore an ECG patch for seven days to confirm a diagnosis of AF. Irregular pulse notifications were received by 0.16% of people aged under 40 but 3.1% of those aged ≥65 years. Of those who received a notification, 18% of people under 40 years were diagnosed with AF but 35% of those aged ≥65 years. If screening is to be both cost effective and clinically relevant, it must be targeted at high-risk groups.

Different types of screening for AF in the population have been suggested, including systematic screening where participants are invited to have an ECG and opportunistic screening where pulse palpation is performed followed by an ECG if an irregular pulse is found.^44–47^ These strategies were informed by randomized trials which used an age threshold for case selection rather than a prediction model with multiple predictors. Our results show that age alone is not the best discriminator of AF risk. Two previous studies also compared risk prediction models to the age criterion of 65 years of age and over and found better discrimination when prediction models were used.^34^^,^^38^

A previous external validation compared nine prediction models to age for predicting the 3-year risk of incident AF using data from the ARIC study. Five models were significantly better than age alone but the CHADS_2_ and CHA_2_DS_2_-VASc scores were not.^38^ We found comparable results of discriminative indices for predicting prevalent AF, indicating that predictors for prevalent and incident AF overlap and the same models might be used for selection of high-risk cases in both situations.

### Strengths and limitations

We conducted a comprehensive literature search to identify all established prediction models, according to a prespecified protocol. We are the first external validation using the outcome prevalent AF, an outcome relevant for a selective screening protocol with a single ECG. A large contemporary screened population of 2.5M participants was used for validation of included models. Included models were validated in the same participants enabling direct comparison of predictive performance. Missing data were handled with multiple imputation and did not affect our findings. Both risk equations and point charts were used for validation if reported. Point charts are easier to apply but contemporary presentation formats, such as webtools and smartphone apps, might use more complicated equations to estimate risks more precisely. We recalibrated risks to update the risk prediction models to the setting of our cohort, with its prevalence of AF.

Most included models were not developed to predict prevalent AF, and this might have influenced predictive performance. Some predictors were not available and for some we used proxies if a direct match was not available which might also have influenced predictive performance. Participants in our cohort were self-referred and self-funded, which might influence generalizability of our findings and might indicate the need to update (the intercept of) the models to new settings before implementation.^26^ Participants were also relatively young and healthy compared to most people who develop AF, which may impact on the external validity of these results to the wider public. Nonetheless, we include data on over 10 000 cases of AF within the population. It is also important to note that studies such as AppleWatch demonstrate a trend to increased screening in younger participants.^43^ Auscultatory or oscillometric sphygmomanometers are recommended in international guidelines to measure SBP and results might have been influenced by using Doppler probes.^48^ Recall bias cannot be excluded for predictors that were self-reported. Symptoms of AF were not recorded. ECG was performed only once in the screened participants, therefore cases of paroxysmal AF are likely to have been missed.^45^ However, given stroke risk increases with frequency of AF, people detected on single-timepoint ECG are more likely to benefit from anticoagulation compared to people with brief episodes of paroxysmal AF, who are most likely to be missed by this approach to screening. Data on use of anticoagulant drugs were not available, but participants with a reported history of AF were excluded from the analyses. The prevalence of AF in our population was lower compared with other populations, possibly making targeted screening more worthwhile in different settings.^6^

### Implications for practice and future research

Recent cohort studies have re-affirmed the importance of using stroke risk assessment tool, such as CHA_2_DS_2_-VASc, to guide anticoagulation decisions and not to withhold this treatment based on high baseline bleeding risk alone.^49^^,^^50^ However, the relatively poor performance of CHA_2_DS_2_-VASc for predicting either AF prevalence or incidence hampers the possibility of using a single score for prediction of AF diagnosis and risk stratification of outcomes, such as stroke or systemic thromboembolism. Using CHA_2_DS_2_-VASc for selection of cases was recently applied by the REHEARSE-AF trial, a randomized controlled trial of AF screening using the AliveCor Kardia smartphone device in people with a CHA_2_DS_2_-VASc score ≥2. Among 1001 participants, 19 were diagnosed with AF in the AliveCor Kardia arm compared to 5 in the control arm at a cost per AF diagnosis of $10 780 in the intervention arm.^51^ Our findings suggest that future research should consider using alternative prediction models, such as CHARGE-AF or MHS to limit screening to high-risk populations and reduce the number needed to screen. Future research will determine how many strokes could be prevented by improved cardiovascular risk management in cases in whom AF is detected by a selective screening programme and whether that leads to a cost-effective screening programme for AF. This might also help determining a threshold probability for selective screening.

Primary care computer software systems currently use electronic alerts based on CHA_2_DS_2_-VASc to help healthcare professionals identify people to consider for opportunistic screening. Such software providers may wish to consider updating their diagnostic algorithms to use a more accurate risk score, such as CHARGE-AF or MHS.

## Conclusions

We identified 14 potential models for predicting prevalent AF, all of which outperformed an age threshold of 65 years, CHADS_2_ and CHA_2_DS_2_-VASc. The CHARGE-AF and MHS risk scores had the highest observed prevalence of AF in the highest decile of predicted risk (1.6%). Using these prediction models could reduce the number needed to screen to detect one case with AF using single time point ECG. Our study showed that established prediction models are able to identify reliably individuals at higher risk of AF. Application of these risk models therefore may be able to inform more selective opportunistic or systematic screening.

## Supplementary material

Supplementary material is available at *European Journal of Preventive Cardiology* online.

## Supplementary Material

zwaa082_Supplementary_Data

## Data Availability

Data from large population-based studies conducted by the Nuffield Department of Population Health can be shared with bona fide researchers on application to the principal investigators of this study. Details of the departmental data access policy can be found at https://www.ndph.ox.ac.uk/data-access.
