# Supplementary material for: Utility of risk prediction models to detect atrial fibrillation in screened participants
Source: Eur J Prev Cardiol. 2020 Nov 29;28(6):586–95. doi: 10.1093/eurjpc/zwaa082 (PMC8651014; doi:10.1093/eurjpc/zwaa082)
Supplement: zwaa082_Supplementary_Data [file zwaa082_supplementary_data.docx]

# eTable 1. Search strategy

|  | MEDLINE (via PubMed interface)^[[1]](#footnote-1)^ |
| --- | --- |
|  | "Atrial fibrillation"[MeSH] OR "Atrial flutter"[MeSH] |
|  | "Atrial fibrillation"[tiab] OR "atrial flutter"[tiab] |
|  | #1 OR #2 |
|  | “Stratification” OR “ROC Curve”[Mesh] OR “Discrimination” OR “Discriminate” OR “c-statistic” OR “c statistic” OR “Area under the curve” OR “AUC” OR “Calibration” OR “Indices” OR “Algorithm” OR “Multivariable” |
|  | "Mass Screening"[MeSH] OR Screen*[tiab] |
|  | Prevalence[MeSH] OR prevalenc*[tiab] OR "incidence"[MeSH Terms] OR incidenc*[tiab] OR communit*[tiab] |
|  | "Population"[MeSH] OR population*[tiab] |
|  | #5 OR #6 OR #7 |
|  | #3 AND #4 AND #8 |
|  |  |
|  | EMBASE (via OVID EMBASE interface)^[[2]](#footnote-2)^ |
|  | exp heart atrium fibrillation/ or exp atrial fibrillation/ or exp heart atrium flutter/ |
|  | (atrial fibrillation or atrial flutter).ti,ab,kw. |
|  | 1 OR 2 |
|  | predict.ti. |
|  | (validat* or rule*).ti,ab. |
|  | (predict* and (outcome* or risk* or model*)).ti,ab. |
|  | ((history or variable* or criteria or scor* or characteristic* or finding* or factor*) and (predict* or model* or decision* or identif* or prognos*)).ti,ab. |
|  | decision*.ti,ab. and statistical model/ |
|  | (decision* and (model* or clinical*)).ti,ab. |
|  | (prognostic and (history or variable* or criteria or scor* or characteristic* or finding* or factor* or model*)).ti,ab. |
|  | (stratification or discrimination or discriminate or c statistic or "area under the curve" or auc or calibration or indices or algorithm or multivariable).ti,ab. |
|  | receiver operating characteristic/ |
|  | 4 OR 5 OR 6 OR 7 OR 8 OR 9 OR 10 OR 11 OR 12 |
|  | exp mass screening/ |
|  | Screening.ab,ti,kw. |
|  | exp prevalence/ |
|  | Prevalence.ab,ti,kw. |
|  | exp incidence/ |
|  | Incidence.ab,ti,kw. |
|  | 14 OR 15 OR 16 OR 17 OR 18 OR 19 |
|  | 3 AND 13 AND 20 |
|  | letter.pt. or letter/ |
|  | note.pt. |
|  | conference abstract.pt. |
|  | editorial.pt. |
|  | case report/ or case study/ |
|  | (letter or comment*).ti. |
|  | 22 OR 23 OR 24 OR 25 OR 26 OR 27 |
|  | animal/ not human/ |
|  | nonhuman/ |
|  | exp animal experiment/ |
|  | exp experimental animal/ |
|  | animal model/ |
|  | exp rodent/ |
|  | (rat or rats or mouse or mice).ti. |
|  | 29 OR 30 OR 31 OR 32 OR 33 OR 34 OR 35 |
|  | 28 OR 36 |
|  | 21 NOT 37 |

# eTable 2. Overview of restrictions applied to inclusion of the original article

|  | **Risk prediction model** | **Restrictions applied to inclusion of the original cohort** |
| --- | --- | --- |
|  | Age >65 years (ESC Guidelines)^1^ | **-** |
|  | Alonso *et al,* 2013 (CHARGE-AF)^2^ ^*^ | **-** |
|  | Aronson *et al,* 2018 (MHS)^3^ | BMI ≥18 & age ≥50 years |
|  | Brunner *et al*, 2014 (MAYO)^4^ | - |
|  | Chamberlain *et al*, 2011 (ARIC)^5^ | Age ≥45 years |
|  | Ding *et al,* 2017 (JINAN)^6^ ^†^ | Age ≥45 years |
|  | Everett et al, 2013 (WHS)^7^ | **-** |
|  | Hamada *et al,* 2019 (SEIREI)^8^ ^‡^ | Age ≥40 years |
|  | Kokubo *et al,* 2017 (SUITA)^9^ | Age ≥30 years |
|  | Li *et al*, 2018 (C_2_HEST)^10^ | **-** |
|  | Linker *et al*, 2018 (SAAFE)^11^ | **-** |
|  | Schnabel *et al,* 2009 (FHS)^12^ | Age ≥45 & ≤84 years |
|  | De Vos *et al*, 2010 (HATCH)^13^ | N/A |
|  | Gage *et al*, 2001 (CHADS_2_)^14^ | N/A |
|  | Lip *et al*, 2010 (CHA_2_DS_2_-VASc)^15^ | N/A |
| ^*^ The ‘simple model’ was assessed; not the ‘augmented model’; ^†^ The ‘simple model’ was assessed; not the ‘ECG model’ and ‘VVV model’; ^‡^ The ‘simple model’ was assessed; not the ‘added model’.  BMI, body mass index; N/A, not applicable. | | |

# eTable 3. Overview of predictors that were not available in our cohort and proxies used

|  | **Risk prediction model** | **Predictors in the risk prediction model that are not relevant for prediction of prevalent AF** | **Predictors not available in our cohort** | **Proxies used** |
| --- | --- | --- | --- | --- |
|  | Age >65 years (ESC Guidelines)^1^ | **-** | **-** | N/A |
|  | Alonso *et al,* 2013 (CHARGE-AF)^2^ | **-** | Race, DBP | Coronary heart disease used for myocardial infarction |
|  | Aronson *et al,* 2018 (MHS)^3^ | - | Female with autoimmune/ inflammatory disease | Coronary heart disease used for myocardial infarction |
|  | Brunner *et al*, 2014 (MAYO)^4^ | - | - | - |
|  | Chamberlain *et al*, 2011 (ARIC)^5^ | Left atrial enlargement, Left ventricular hypertrophy | Race | Valvular disease used for precordial murmur |
|  | Ding *et al,* 2017 (JINAN)^6^ | - | **-** | - |
|  | Everett et al, 2013 (WHS)^7^ | **-** | Alcohol drinking status | - |
|  | Hamada *et al,* 2019 (SEIREI)^8^ | - | WC, DBP, Alcohol drinking status | Valvular disease used for cardiac murmur |
|  | Kokubo *et al,* 2017 (SUITA)^9^ | non-HDL-C | Arrhythmia, Alcohol drinking status | Valvular disease used for cardiac murmur |
|  | Li *et al*, 2018 (C_2_HEST)^10^ | **-** | Hyperthyroidism | Congestive heart failure used for systolic heart failure |
|  | Linker *et al*, 2018 (SAAFE)^11^ | **-** | Cardiac arrest, Kidney transplant, Hospitalized, Coronary artery stent, Race | - |
|  | Schnabel *et al,* 2009 (FHS)^12^ | **-** | **-** | Valvular disease used for significant murmur |
|  | De Vos *et al*, 2010 (HATCH)^13^ | **-** | **-** | - |
|  | Gage *et al*, 2001 (CHADS_2_)^14^ | **-** | **-** | N/A |
|  | Lip *et al*, 2010 (CHA_2_DS_2_-VASc)^15^ | **-** | **-** | N/A |
| AF, atrial fibrillation; DBP, diastolic blood pressure, HDL-C, High-density lipoprotein-cholesterol; N/A not applicable; WC, waist circumference. | | | | |

# eTable 4. Missing data in our cohort

| **Variables included as predictors** | **Percentage of participants with missing in our cohort** |
| --- | --- |
| Age | 0 |
| Sex | 0 |
| SBP | 0.7 |
| Heart rate | 0.8 |
| PAD | 1.6 |
| Height | 1.8 |
| Weight | 2.8 |
| BMI | 3.9 |
| Hypertension | 4.5 |
| Hypercholesterolemia | 5.4 |
| CHF | 6.7 |
| Antihypertensive therapy | 7.2 |
| DM | 8.5 |
| Smoking status | 10.7 |
| CHD | 12.2 |
| Stroke or TIA | 13.4 |
| Valvular disease | 24.6 |
| COPD | 25.2 |
| BMI, body mass index; CHD, coronary heart disease; CHF, congestive heart failure; COPD, chronic obstructive pulmonary disease; DM, diabetes mellitus; PAD, peripheral arterial disease; SBP, systolic blood pressure; TIA, transient ischemic attack. | |

# eTable 5. Calculation of linear predictor functions

|  | **Risk prediction model** | **Calculation of linear predictor for present analyses** |
| --- | --- | --- |
|  | Age >65 years (ESC Guidelines)^1^ | - |
|  | Alonso *et al,* 2013 (CHARGE-AF)^2^ | $LP_{\mathrm{Alonso}}=\left( 0.1016\times Age \right)+\left( 0.0248\times Height [per cm] \right)+\left( 0.007667\times Weight [per kg] \right)+\left( 0.00985 \times SBP [per mmHg] \right)+\left( 0.359\times Current smoking \right)+\left( 0.349\times Antihypertensives \right)+\left( 0.237\times Diabetes \right)+\left( 0.701\times Congestive heart failure \right)+(0.496\times Coronary heart disease)$ |
|  | Aronson *et al,* 2018 (MHS)^3^ | $\mathrm{LP}_{\mathrm{Aronson}}=\left( 0.0698\times Age \right)+(0.0492\times BMI [per kg/m^{2}])+\left( -0.370\times Female sex \right)+\left( 0.390 \times Treated hypertension \right)+(-0.063\times Age in patients with congestive heart feailure)+(0.2\times History of stroke or peripheral arterial disease)+(5.25*History of congestive heart failure)+(0.20\times History of COPD)+(0.305\times History of MI)+ (0.24\times SBP \geq160 mmHg)$ |
|  | Brunner *et al*, 2014 (MAYO)^4^ | $\mathrm{LP}_{\mathrm{Brunner}}=\left( 1.28\times Heart failure \right)+\left( 0.88\times Valvular disease \right)+\left( 0.74\times Coronary heart disease \right)+\left( 0.074\times Age \right)+(0.47\times Hypertension)+(0.47\times Diabetes)+(0.41\times Male sex)$ |
|  | Chamberlain *et al*, 2011 (ARIC)^5^ | $LP_{\mathrm{Chamberlain}}= \left( 0 \times Age 45-49 \right)+ \left( 0.6307 \times Age 50-54 \right)+ \left( 0.97608 \times Age 55-59 \right)+ \left( 1.70756\times Age \geq60-64 \right)+\left( 0 \times Height < 1.64m \right)+ \left( 0.31422 \times Height 1.64-1.72m \right)+ \left( 0.80714 \times Height \geq1.73m \right)+ \left( 0.01138\times SBP \left[ \mathrm{mmHg} \right] \right)+\left( 0.62318\times Antihypertensives \right)+\left( 0\times Never smoking \right)+\left( 0.19445\times Former smoking \right)+\left( 0.57045\times Current smoking \right) +\left( 0.43563\times Valvular disease \right)+\left( 0.8992*Diabetes \right)+ \left( 0\times Diabetes at age 45-50 \right)+\left( 0.07979\times Diabetes at age 50-55 \right)+\left( -0.59912\times Diabetes at age 55-60 \right)+\left( -0.85881\times Diabetes at age \geq60 \right)+\left( 0.46524\times CHF \right)+\left( 1.23089\times CHD \right)+\left( 0\times CHD at age<45 \right)+ \left( 0\times CHD at age 45-49 \right)+\left( -0.66455\times CHD at age 50-54 \right)+\left( -0.52404\times CHD at age 55-59 \right)+(-1.19565\times CHD at age \geq60)$ |
|  | Ding *et al,* 2017 (JINAN)^6*^ | $LP_{\mathrm{Ding}}=(0.07139*Age)+\left( 0.741*Male sex \right)+\left( 0.633\times Coronary heart disease \right)+(0.449\times Hypertension)$ |
|  | Everett et al, 2013 (WHS)^7^ | $LP_{\mathrm{Everett}}=\left( 0.5480\times\ln\left( \mathrm{Age} \right) \right)+\left( 0.0157\times Weight [per 1 kg] \right)+\left( 0.0306\times Height [per 1 cm] \right)+\left( 0.0155\times SBP [per 1 mmHg] \right)+(0.254\times Ever smoker)$ |
|  | Hamada *et al,* 2019 (SEIREI)^8^ | $LP_{\mathrm{Hamada}}=(0.197 \times Age 45-49) + (1.119\times Age 50-54) + (1.233 \times Age 55-59) + (1.885 \times Age 60-64) + (1.997 \times Age 65-69) + (2.632 \times Age 70-74) + (2.227 \times Age 75-79) + (0.687 \times Male sex) + (0 \times HR > 50/min) + (0.674 \times HR \leq50/min) + (0.934 \times History of valvular disease)$ |
|  | Kokubo *et al,* 2017 (SUITA)^9^ | $LP_{\mathrm{Kokubo}}=\left( 0\times Age<50 in men \right)+\left( 0.871\times Age 50-59 in men \right)+\left( 1.860\times Age 60-69 in men \right)+\left( 2.428\times Age \geq70 in men \right)+\left( -1.329\times Age<50 in women \right)+\left( -0.198\times Age50-59 in women \right)+\left( 1.410\times Age 60-69 in women \right)+\left( 2.434\times Age \geq70 \right)+\left( 0\times SBP<120 mmHg \right)+\left( 0.297\times SBP 120-139 mmHg \right)+\left( 0.544\times SBP\geq140 mmHg \right)+\left( 0.297\times Antihypertensives \right)+\left( 0\times BMI<25 kg/m^{2} \right)+(0.427\times BMI\geq25 kg/m^{2}) +(0.336\times Current smoking)+(0.606\times Coronary heart disease)+(2.209\times Valvular disease at age 30-49)+(1.720\times Valvular disease at age 50-59)+(0.607\times Valvular disase at age 60-69)+(0\times Valvular disease at age \geq70)$ |
|  | Li *et al*, 2018 (C_2_HEST)^10^ | $LP_{\mathrm{Li}}=\left( 1.421\times Coronary heart disease \right)+\left( 1.102\times COPD \right)+\left( 1.176\times Hypertension \right)+\left( 1.763\times Age\geq75 \right)+(2.073\times Congestive heart failure)$ |
|  | Linker *et al*, 2018 (SAAFE)^11^ | $LP_{\mathrm{Linker}}=\left( 0.023\times Age \right)+\left( 0.68\times Height \left[ m \right] \right)+(0.25\times(Height [m]\times Weight [kg]/100))+(0.78\times Congestive heart failure)+(0.37\times Coronary artery disease)+(0.30\times COPD)+(0.12\times Stroke)+(-0.06\times Diabetes)$ |
|  | Schnabel *et al,* 2009 (FHS)^12^ | $LP_{\mathrm{Schnabel}}=\left( 0.14655\times Age \right)+\left( -0.0003275\times\mathrm{Age}^{2} \right)+\left( 2.05393\times Male sex \right)+(0.02130\times BMI[kg/m^{2}])+(0.00575\times SBP \left[ \mathrm{mmHg} \right])+(0.47093\times Antihypertensives)+(4.18531\times Valvular disease)+(9.89758\times Congestive heart failure)+(-0.0002774\times\mathrm{age}^{2}in males)+(-0.04743\times Age in patients with valvular disease)+(-0.12776\times Age in patients with congestive heart failure)$ |
|  | De Vos *et al*, 2010 (HATCH)^13^ | $LP_{de Vos}=\left( 0.80\times Congestive heart failure \right)+\left( 0.42\times Hypertension \right)+\left( 0.41\times COPD \right)+\left( 0.71\times Stroke \right)+(0.45\times Age>75)$ |
|  | Gage *et al*, 2001 (CHADS_2_)^14^ | - |
|  | Lip *et al*, 2010 (CHA_2_DS_2_-VASc)^15^ | - |

# eTable 6. Full-text evaluation

|  | **Reason for exclusion** | **Number of studies** |
| --- | --- | --- |
|  | Selected population/ population at high risk for AF | 23^16-38^ |
|  | AF as domain | 1^39^ |
|  | Review on prediction of AF | 5^40-44^ |
|  | Other reviews | 19^45-63^ |
|  | Initiation or evaluation of screening program/method | 17^64-80^ |
|  | Health-economic research | 7^81-87^ |
|  | Prevalence of AF estimated without prediction model | 22^88-109^ |
|  | Etiologic research: determination of risk factors for AF | 55^110-164^ |
|  | Etiologic research: AF as risk factor | 2^165, 166^ |
|  | Diagnostic research | 26^167-192^ |
|  | Prognostic research: predicted outcome is risk of stroke and /or antithrombotic treatment | 16^193-208^ |
|  | Prognostic research: different predicted outcome (or AF not separately provided) | 4^209-212^ |
|  | Prognostic research: different determinant (not ECG) | 4^213-216^ |
|  | Article retracted | 1^217^ |
|  | External validation study | 16^218-233^ |
|  | Incremental/added value study | 13^234-246^ |
|  | Inclusion of genetic predictors | 3^247-249^ |
| AF, atrial fibrillation; ECG, electrocardiogram | | |

# eTable 7. Discriminative performance in all participants and participants with CHA_2_DS_2_-VASc of 2 or more

|  |  | **All participants**  **(N = 2,541,702)** | | **Participants with CHA_2_DS_2_-VASc of 2 or more**  **(N = 1,153,878)** | |
| --- | --- | --- | --- | --- | --- |
|  | **Risk prediction model** | **AUROC curve (95% CI) of regression equation** | **AUROC curve (95% CI) of point chart** | **AUROC curve (95% CI) of regression equation** | **AUROC curve (95% CI) of point chart** |
|  | Age >65 years (ESC Guidelines)^1^ | 0.655 (0.651-0.658) | N/A | 0.585 (0.579-0.592) | N/A |
|  | Alonso *et al,* 2013 (CHARGE-AF)^2^ | 0.764 (0.759-0.768) | N/A | 0.749 (0.743-0.755) | N/A |
|  | Aronson *et al,* 2018 (MHS)^3^ | 0.762 (0.757-0.766) | 0.756 (0.752-0.761) | 0.748 (0.742-0.755) | 0.740 (0.733-0.746) |
|  | Brunner *et al*, 2014 (MAYO)^4^ | 0.747 (0.743-0.752) | 0.724 (0.719-0.728) | 0.729 (0.722-0.735) | 0.693 (0.686-0.699) |
|  | Chamberlain *et al*, 2011 (ARIC)^5^ | 0.693 (0.688-0.698) | 0.694 (0.689-0.699) | 0.649 (0.642-0.656) | 0.651 (0.643-0.658) |
|  | Ding *et al,* 2017 (JINAN)^6^ | 0.758 (0.753-0.762) | 0.754 (0.750-0.759) | 0.737 (0.731-0.744) | 0.732 (0.725-0.738) |
|  | Everett et al, 2013 (WHS)^7^ | 0.672 (0.667-0.678) | N/A | 0.664 (0.656-0.671) | N/A |
|  | Hamada *et al,* 2019 (SEIREI)^8^ | 0.716 (0.711-0.720) | 0.734 (0.730-0.739) | 0.681 (0.674-0.687) | 0.704 (0.698-0.711) |
|  | Kokubo *et al,* 2017 (SUITA)^9^ | 0.714 (0.710-0.719) | 0.714 (0.709-0.718) | 0.666 (0.659-0.672) | 0.667 (0.661-0.673) |
|  | Li *et al*, 2018 (C_2_HEST)^10^ | 0.685 (0.679-0.690) | 0.686 (0.681-0.691) | 0.675 (0.667-0.682) | 0.676 (0.669-0.684) |
|  | Linker *et al*, 2018 (SAAFE)^11^ | 0.771 (0.767-0.776) | N/A | 0.750 (0.744-0.757) | N/A |
|  | Schnabel *et al,* 2009 (FHS)^12^ | 0.756 (0.752-0.761) | 0.757 (0.753-0.762) | 0.732 (0.726-0.739) | 0.733 (0.726-0.739) |
|  | De Vos *et al*, 2010 (HATCH)^13^ | 0.680 (0.675-0.685) | 0.661 (0.656-0.667) | 0.667 (0.659-0.674) | 0.632 (0.625-0.639) |
|  | Gage *et al*, 2001 (CHADS_2_)^14^ | N/A | 0.658 (0.653-0.664) | N/A | 0.625 (0.618-0.632) |
|  | Lip *et al*, 2010 (CHA_2_DS_2_-VASc)^15^ | N/A | 0.650 (0.645-0.656) | N/A | 0.608 (0.600-0.616) |
| AUROC, area under receiver operating characteristic curve; CI, confidence interval; N/A, not applicable. | | | | | |

# eTable 8. Sensitivity analyses: Discriminative performance in complete cases

|  | **Risk prediction model** | **AUROC curve (95% CI) of regression equation** | **N participants** | **AUROC curve (95% CI) of point chart** | **N participants** |
| --- | --- | --- | --- | --- | --- |
|  | Age >65 years (ESC Guidelines)^1^ | 0.655 (0.651-0.658) | 2,541,702 | N/A | - |
|  | Alonso *et al,* 2013 (CHARGE-AF)^2^ | 0.756 (0.750-0.763) | 1,699,577 | N/A | - |
|  | Aronson *et al,* 2018 (MHS)^3^ | 0.753 (0.746-0.759) | 1,730,244^*^ | 0.744 (0.738-0.750) | 1,737,641^*^ |
|  | Brunner *et al*, 2014 (MAYO)^4^ | 0.738 (0.732-0.744) | 1,818,258 | 0.715 (0.709-0.720) | 1,818,258 |
|  | Chamberlain *et al*, 2011 (ARIC)^5^ | 0.684 (0.678-0.691) | 1,543,859 | 0.686 (0.679-0.692) | 1,543,859 |
|  | Ding *et al,* 2017 (JINAN)^6^ | 0.751 (0.746-0.756) | 2,185,490 | 0.748 (0.743-0.753) | 2,185,490 |
|  | Everett et al, 2013 (WHS)^7^ | 0.672 (0.666-0.678) | 2,180,155 | N/A | - |
|  | Hamada *et al,* 2019 (SEIREI)^8^ | 0.712 (0.707-0.718) | 1,901,920 | 0.732 (0.726-0.737) | 1,901,920 |
|  | Kokubo *et al,* 2017 (SUITA)^9^ | 0.706 (0.700-0.712) | 1,574,604^†^ | 0.706 (0.700-0.712) | 1,590,084^†^ |
|  | Li *et al*, 2018 (C_2_HEST)^10^ | 0.676 (0.669-0.682) | 1,847,294 | 0.677 (0.670-0.684) | 1,847,294 |
|  | Linker *et al*, 2018 (SAAFE)^11^ | 0.765 (0.759-0.770) | 1,778,013 | N/A | - |
|  | Schnabel *et al,* 2009 (FHS)^12^ | 0.751 (0.746-0.757) | 1,786,232 | 0.752 (0.746-0.758) | 1,786,232 |
|  | De Vos *et al*, 2010 (HATCH)^13^ | 0.671 (0.664-0.677) | 1,850,039 | 0.651 (0.646-0.658) | 1,850,039 |
|  | Gage *et al*, 2001 (CHADS_2_)^14^ | N/A | - | 0.652 (0.646-0.658) | 2,031,416 |
|  | Lip *et al*, 2010 (CHA_2_DS_2_-VASc)^15^ | N/A | - | 0.640 (0.634-0.647) | 1,933,625 |
| ^*^ The number of participants differed between the point chart and regression equation of the MHS prediction model by Aronson *et al*, 2018, because CVA was included as predictor in the regression equation but not in the point chart. ^†^ The number of participants differed between the point chart and regression equation of the SUITA prediction model by Kokubo *et al*, 2017, because antihypertensive therapy was included as predictor in the regression equation but not in the point chart.  AUROC, area under receiver operating characteristic curve; CI, confidence interval; N, number; N/A, not applicable. | | | | | |

# eTable 9. Predicted and observed prevalence of atrial fibrillation across deciles or groups of predicted risk

## Alonso et al, 2013 (CHARGE-AF)^2^

|  | Deciles of predicted risk of atrial fibrillation (AF) | | | | | | | | | |
| --- | --- | --- | --- | --- | --- | --- | --- | --- | --- | --- |
| Predicted prevalence based on risk prediction model (%) | 0.03 | 0.06 | 0.09 | 0.12 | 0.17 | 0.24 | 0.33 | 0.48 | 0.76 | 1.78 |
| Number of cases with AF | 148 | 232 | 262 | 334 | 568 | 627 | 977 | 1289 | 1938 | 4089 |
| Observed prevalence of AF (%) | 0.06 | 0.08 | 0.11 | 0.13 | 0.20 | 0.27 | 0.36 | 0.50 | 0.75 | 1.59 |

## Aronson et al, 2018 (MHS)^3^

|  | Deciles of predicted risk of atrial fibrillation (AF) | | | | | | | | | |
| --- | --- | --- | --- | --- | --- | --- | --- | --- | --- | --- |
| Predicted prevalence based on risk prediction model (%) | 0.07 | 0.12 | 0.15 | 0.19 | 0.24 | 0.31 | 0.40 | 0.52 | 0.72 | 1.33 |
| Number of cases with AF | 150 | 224 | 284 | 313 | 521 | 722 | 955 | 1291 | 1891 | 4113 |
| Observed prevalence of AF (%) | 0.06 | 0.09 | 0.11 | 0.15 | 0.19 | 0.26 | 0.37 | 0.50 | 0.75 | 1.58 |

## Brunner et al, 2014 (MAYO)^4^

|  | Deciles of predicted risk of atrial fibrillation (AF) | | | | | | | | | |
| --- | --- | --- | --- | --- | --- | --- | --- | --- | --- | --- |
| Predicted prevalence based on risk prediction model (%) | 0.06 | 0.09 | 0.12 | 0.16 | 0.21 | 0.27 | 0.35 | 0.47 | 0.67 | 1.66 |
| Number of cases with AF | 151 | 218 | 346 | 412 | 523 | 787 | 939 | 1276 | 1878 | 3934 |
| Observed prevalence of AF (%) | 0.07 | 0.09 | 0.12 | 0.17 | 0.20 | 0.29 | 0.37 | 0.51 | 0.73 | 1.52 |

## Chamberlain et al, 2011 (ARIC)^5^

|  | Deciles of predicted risk of atrial fibrillation (AF) | | | | | | | | | |
| --- | --- | --- | --- | --- | --- | --- | --- | --- | --- | --- |
| Predicted prevalence based on risk prediction model (%) | 0.07 | 0.13 | 0.18 | 0.23 | 0.30 | 0.37 | 0.44 | 0.53 | 0.70 | 1.13 |
| Number of cases with AF | 153 | 257 | 420 | 641 | 789 | 1058 | 1055 | 1471 | 1923 | 2697 |
| Observed prevalence of AF (%) | 0.07 | 0.11 | 0.16 | 0.23 | 0.31 | 0.42 | 0.46 | 0.55 | 0.72 | 1.06 |

## Ding et al, 2017 (JINAN)^6^

|  | Deciles of predicted risk of atrial fibrillation (AF) | | | | | | | | | |
| --- | --- | --- | --- | --- | --- | --- | --- | --- | --- | --- |
| Predicted prevalence based on risk prediction model (%) | 0.06 | 0.10 | 0.14 | 0.19 | 0.24 | 0.30 | 0.38 | 0.51 | 0.71 | 1.40 |
| Number of cases with AF | 117 | 232 | 303 | 411 | 455 | 726 | 1034 | 1248 | 1963 | 3975 |
| Observed prevalence of AF (%) | 0.06 | 0.08 | 0.11 | 0.16 | 0.19 | 0.29 | 0.37 | 0.50 | 0.77 | 1.51 |

## Everett et al, 2013 (WHS)^7^

|  | Deciles of predicted risk of atrial fibrillation (AF) | | | | | | | | | |
| --- | --- | --- | --- | --- | --- | --- | --- | --- | --- | --- |
| Predicted prevalence based on risk prediction model (%) | 0.12 | 0.17 | 0.21 | 0.24 | 0.29 | 0.34 | 0.41 | 0.51 | 0.65 | 1.12 |
| Number of cases with AF | 274 | 380 | 553 | 590 | 824 | 956 | 1150 | 1275 | 1665 | 2797 |
| Observed prevalence of AF (%) | 0.13 | 0.16 | 0.21 | 0.24 | 0.30 | 0.36 | 0.42 | 0.50 | 0.67 | 1.07 |

## Hamada et al, 2019 (SEIREI)^8^

|  | Deciles of predicted risk of atrial fibrillation (AF) | | | | | | | | | |
| --- | --- | --- | --- | --- | --- | --- | --- | --- | --- | --- |
| Predicted prevalence based on risk prediction model (%) | 0.10 | 0.13 | 0.24 | 0.26 | 0.28 | 0.30 | 0.42 | 0.54 | 0.59 | 1.01 |
| Number of cases with AF | 176 | 218 | 173 | 592 | 52 | 606 | 2599 | 609 | 1190 | 4249 |
| Observed prevalence of AF (%) | 0.07 | 0.09 | 0.16 | 0.15 | 0.17 | 0.19 | 0.60 | 0.28 | 0.58 | 1.25 |

## Kokubo et al, 2017 (SUITA)^9^

|  | Deciles of predicted risk of atrial fibrillation (AF) | | | | | | | | | |
| --- | --- | --- | --- | --- | --- | --- | --- | --- | --- | --- |
| Predicted prevalence based on risk prediction model (%) | 0.02 | 0.06 | 0.12 | 0.19 | 0.25 | 0.34 | 0.45 | 0.61 | 0.80 | 1.18 |
| Number of cases with AF | 117 | 269 | 357 | 403 | 407 | 1023 | 1298 | 1835 | 2075 | 2680 |
| Observed prevalence of AF (%) | 0.06 | 0.09 | 0.15 | 0.17 | 0.21 | 0.30 | 0.56 | 0.66 | 0.80 | 1.04 |

## Li et al, 2018 (C_2_HEST)^10^

|  | Groups of predicted risk of atrial fibrillation (AF) | | | |
| --- | --- | --- | --- | --- |
| Predicted prevalence based on risk prediction model (%) | 0.07 | 0.22 | 0.53 | 2.42 |
| Number of cases with AF | 2587 | 2240 | 2317 | 3320 |
| Observed prevalence of AF (%) | 0.21 | 0.29 | 0.86 | 1.25 |

## Linker et al, 2018 (SAAFE)^11^

|  | Deciles of predicted risk of atrial fibrillation (AF) | | | | | | | | | | |
| --- | --- | --- | --- | --- | --- | --- | --- | --- | --- | --- | --- |
| Predicted prevalence based on risk prediction model (%) | 0.25 | 0.29 | 0.32 | 0.34 | 0.36 | 0.39 | 0.42 | 0.46 | 0.52 | 0.71 |  |
| Number of cases with AF | 136 | 199 | 174 | 372 | 336 | 628 | 1068 | 1162 | 1940 | 4449 |  |
| Observed prevalence of AF (%) | 0.06 | 0.08 | 0.09 | 0.12 | 0.17 | 0.23 | 0.34 | 0.49 | 0.75 | 1.62 |  |

## Schnabel et al, 2009 (FHS)^12^

|  | Deciles of predicted risk of atrial fibrillation (AF) | | | | | | | | | | |
| --- | --- | --- | --- | --- | --- | --- | --- | --- | --- | --- | --- |
| Predicted prevalence based on risk prediction model (%) | 0.03 | 0.06 | 0.11 | 0.16 | 0.22 | 0.30 | 0.40 | 0.55 | 0.78 | 1.44 |  |
| Number of cases with AF | 123 | 167 | 307 | 334 | 530 | 693 | 1004 | 1394 | 2144 | 3768 |  |
| Observed prevalence of AF (%) | 0.06 | 0.07 | 0.11 | 0.14 | 0.20 | 0.27 | 0.40 | 0.54 | 0.83 | 1.45 |  |

## de Vos et al, 2010 (HATCH)^13^

|  | Groups of predicted risk of atrial fibrillation (AF) | | | | |
| --- | --- | --- | --- | --- | --- |
| Predicted prevalence based on risk prediction model (%) | 0.29 | 0.43 | 0.44 | 0.49 | 0.84 |
| Number of cases with AF | 2714 | 130 | 1875 | 2493 | 3252 |
| Observed prevalence of AF (%) | 0.21 | 0.41 | 0.28 | 0.81 | 1.24 |

# eTable 10. Performance of the risk prediction models to detect atrial fibrillation

| **Targeted screening** | **Number of cases with AF** | **Number of individuals** | **Observed prevalence** | **Sensitivity** | **Specificity** | **PPV** | **NPV** | **NNS** |
| --- | --- | --- | --- | --- | --- | --- | --- | --- |
| ***Highest decile of predicted risk*** | | | | | | | | |
| Alonso *et al*, 2013 (CHARGE-AF)^2^ | 4089 | 257,309 | 1.6 | 39.1 | 90.0 | 1.6 | 99.7 | 63 |
| Aronson *et al*, 2018 (MHS)^3^ | 4113 | 259,499 | 1.6 | 39.3 | 89.9 | 1.6 | 99.7 | 63 |
| Brunner *et al*, 2014 (MAYO)^4^ | 3934 | 259,377 | 1.5 | 37.6 | 89.9 | 1.5 | 99.7 | 66 |
| Chamberlain *et al*, 2011 (ARIC)^5^ | 2697 | 254,709 | 1.1 | 25.8 | 90.0 | 1.1 | 99.7 | 94 |
| Ding *et al*, 2017 (JINAN)^6^ | 3975 | 263,167 | 1.5 | 38.0 | 89.8 | 1.5 | 99.7 | 66 |
| Everett *et al*, 2013 (WHS)^7^ | 2797 | 262,410 | 1.1 | 26.7 | 89.7 | 1.1 | 99.7 | 94 |
| Hamada *et al*, 2018 (SEIREI)^8^ | 4249 | 340,015 | 1.3 | 40.6 | 86.7 | 1.2 | 99.7 | 80 |
| Kokubo *et al*, 2017 (SUITA)^9^ | 2680 | 257,586 | 1.0 | 25.6 | 89.9 | 1.0 | 99.7 | 96 |
| Li *et al*, 2018 (C_2_HEST)^10^ | 3320 | 266,364 | 1.3 | 31.7 | 89.6 | 1.2 | 99.7 | 80 |
| Linker *et al*, 2018 (SAAFE)^11^ | 4449 | 275,437 | 1.6 | 42.5 | 89.3 | 1.6 | 99.7 | 62 |
| Schnabel *et al*, 2009 (FHS)^12^ | 3768 | 259,529 | 1.5 | 36.0 | 89.9 | 1.5 | 99.7 | 69 |
| de Vos *et al*, 2010 (HATCH)^13^ | 3252 | 262,806 | 1.2 | 31.1 | 89.7 | 1.2 | 99.7 | 81 |
| ***Highest two deciles of predicted risk*** | | | | | | | | |
| Alonso *et al*, 2013 (CHARGE-AF)^2^ | 6027 | 515,724 | 1.3 | 48.3 | 84.9 | 1.3 | 99.7 | 76 |
| Aronson *et al*, 2018 (MHS)^3^ | 6004 | 511,979 | 1.3 | 48.3 | 85.0 | 1.3 | 99.7 | 76 |
| Brunner *et al*, 2014 (MAYO)^4^ | 5812 | 514,891 | 1.3 | 46.6 | 84.9 | 1.3 | 99.7 | 79 |
| Chamberlain *et al*, 2011 (ARIC)^5^ | 4620 | 523,354 | 0.9 | 35.0 | 84.8 | 0.9 | 99.7 | 106 |
| Ding *et al*, 2017 (JINAN)^6^ | 5938 | 518,147 | 1.3 | 47.4 | 84.8 | 1.3 | 99.7 | 79 |
| Everett *et al*, 2013 (WHS)^7^ | 4462 | 511,262 | 0.9 | 34.7 | 84.9 | 0.9 | 99.7 | 107 |
| Hamada *et al*, 2019 (SEIREI)^8^ | 5439 | 544,824 | 1.1 | 46.3 | 82.7 | 1.1 | 99.7 | 91 |
| Kokubo *et al*, 2017 (SUITA)^9^ | 4755 | 517,322 | 0.9 | 35.5 | 84.8 | 1.0 | 99.7 | 104 |
| Li *et al*, 2018 (C_2_HEST)^10^ | 5637 | 537,327 | 1.1 | 42.8 | 84.3 | 1.1 | 99.7 | 90 |
| Linker *et al*, 2018 (SAAFE)^11^ | 6389 | 532,561 | 1.3 | 51.8 | 84.3 | 1.3 | 99.8 | 75 |
| Schnabel *et al*, 2009 (FHS)^12^ | 5912 | 518,216 | 1.2 | 46.3 | 84.8 | 1.2 | 99.7 | 80 |
| de Vos *et al*, 2010 (HATCH)^13^ | 5745 | 572,249 | 1.1 | 43.0 | 83.7 | 1.1 | 99.7 | 93 |
| AF, Atrial fibrillation; NNS, number needed to screen; NPV, negative predictive value; PPV, positive predictive value. | | | | | | | | |

# eFigure 1. Calibration plots

Calibration plots. Data of 2.5M participants was used to construct these calibration plots. Mean predicted risk against the observed risk of AF across deciles or groups of predicted risk (after recalibration with adjusting the intercept) is shown. The boxes represent the mean predicted risk for each decile or group and the vertical lines represent the 95% confidence intervals. The dotted diagonal line indicates perfect calibration. Boxes above the diagonal line indicate underestimation of risk and below the diagonal line overestimation of risk. The prevalences and number of cases per decile or group are provided in eTable 8.

# eFigure 2. Observed prevalences by sum scores, using the risk scores

# References

1. Kirchhof P, Benussi S, Kotecha D, Ahlsson A, Atar D, Casadei B, Castella M, Diener HC, Heidbuchel H, Hendriks J, Hindricks G, Manolis AS, Oldgren J, Popescu BA, Schotten U, Van Putte B, Vardas P and Group ESCSD. 2016 ESC Guidelines for the management of atrial fibrillation developed in collaboration with EACTS. *Eur Heart J*. 2016;37:2893-2962.

2. Alonso A, Krijthe BP, Aspelund T, Stepas KA, Pencina MJ, Moser CB, Sinner MF, Sotoodehnia N, Fontes JD, Janssens AC, Kronmal RA, Magnani JW, Witteman JC, Chamberlain AM, Lubitz SA, Schnabel RB, Agarwal SK, McManus DD, Ellinor PT, Larson MG, Burke GL, Launer LJ, Hofman A, Levy D, Gottdiener JS, Kaab S, Couper D, Harris TB, Soliman EZ, Stricker BH, Gudnason V, Heckbert SR and Benjamin EJ. Simple risk model predicts incidence of atrial fibrillation in a racially and geographically diverse population: the CHARGE-AF consortium. *J Am Heart Assoc*. 2013;2:e000102.

3. Aronson D, Shalev V, Katz R, Chodick G and Mutlak D. Risk Score for Prediction of 10-Year Atrial Fibrillation: A Community-Based Study. *Thromb Haemost*. 2018;118:1556-1563.

4. Brunner KJ, Bunch TJ, Mullin CM, May HT, Bair TL, Elliot DW, Anderson JL and Mahapatra S. Clinical predictors of risk for atrial fibrillation: implications for diagnosis and monitoring. *Mayo Clin Proc*. 2014;89:1498-505.

5. Chamberlain AM, Agarwal SK, Folsom AR, Soliman EZ, Chambless LE, Crow R, Ambrose M and Alonso A. A clinical risk score for atrial fibrillation in a biracial prospective cohort (from the Atherosclerosis Risk in Communities [ARIC] study). *Am J Cardiol*. 2011;107:85-91.

6. Ding L, Li J, Wang C, Li X, Su Q, Zhang G and Xue F. Incidence of atrial fibrillation and its risk prediction model based on a prospective urban Han Chinese cohort. *J Hum Hypertens*. 2017;31:574-579.

7. Everett BM, Cook NR, Conen D, Chasman DI, Ridker PM and Albert CM. Novel genetic markers improve measures of atrial fibrillation risk prediction. *Eur Heart J*. 2013;34:2243-51.

8. Hamada R and Muto S. Simple risk model and score for predicting of incident atrial fibrillation in Japanese. *J Cardiol*. 2019;71:65-72.

9. Kokubo Y, Watanabe M, Higashiyama A, Nakao YM, Kusano K and Miyamoto Y. Development of a Basic Risk Score for Incident Atrial Fibrillation in a Japanese General Population- The Suita Study. *Circ J*. 2017;81:1580-1588.

10. Li YG, Pastori D, Farcomeni A, Yang PS, Jang E, Joung B, Wang YT, Guo YT and Lip GYH. A simple clinical risk score (C2HEST) for predicting incident atrial fibrillation in Asian subjects: Derivation in 471,446 Chinese subjects, with internal validation and external application in 451,199 Korean subjects. *Chest*. 2018;261:92-8.

11. Linker DT, Murphy TB and Mokdad AH. Selective screening for atrial fibrillation using multivariable risk models. *Heart*. 2018;104:1492-1499.

12. Schnabel RB, Sullivan LM, Levy D, Pencina MJ, Massaro JM, D'Agostino RB, Sr., Newton-Cheh C, Yamamoto JF, Magnani JW, Tadros TM, Kannel WB, Wang TJ, Ellinor PT, Wolf PA, Vasan RS and Benjamin EJ. Development of a risk score for atrial fibrillation (Framingham Heart Study): a community-based cohort study. *Lancet*. 2009;373:739-45.

13. de Vos CB, Pisters R, Nieuwlaat R, Prins MH, Tieleman RG, Coelen RJ, van den Heijkant AC, Allessie MA and Crijns HJ. Progression from paroxysmal to persistent atrial fibrillation clinical correlates and prognosis. *J Am Coll Cardiol*. 2010;55:725-31.

14. Gage BF, Waterman AD, Shannon W, Boechler M, Rich MW and Radford MJ. Validation of clinical classification schemes for predicting stroke: results from the National Registry of Atrial Fibrillation. *JAMA*. 2001;285:2864-70.

15. Lip GY, Nieuwlaat R, Pisters R, Lane DA and Crijns HJ. Refining clinical risk stratification for predicting stroke and thromboembolism in atrial fibrillation using a novel risk factor-based approach: the euro heart survey on atrial fibrillation. *Chest*. 2010;137:263-72.

16. Abdulla J and Nielsen JR. Is the risk of atrial fibrillation higher in athletes than in the general population? A systematic review and meta-analysis. *Europace*. 2009;11:1156-9.

17. Alkhouli M, Alqahtani F, Aljohani S, Alvi M and Holmes DR. Burden of Atrial Fibrillation-Associated Ischemic Stroke in the United States. *JACC: Clinical Electrophysiology*. 2018;4:618-625.

18. Barrett TW, Couch SA, Jenkins CA and Storrow AB. Prevalence of validated risk factors for developing atrial fibrillation - Can we identify high-risk ED patients? *American Journal of Emergency Medicine*. 2012;30:1581-1587.

19. Barrett TW, Jenkins CA and Self WH. Validation of the Risk Estimator Decision Aid for Atrial Fibrillation (RED-AF) for predicting 30-day adverse events in emergency department patients with atrial fibrillation. *Ann Emerg Med*. 2015;65:13-21.

20. Barrett TW, Self WH, Wasserman BS, McNaughton CD and Darbar D. Evaluating the HATCH score for predicting progression to sustained atrial fibrillation in ED patients with new atrial fibrillation. *Am J Emerg Med*. 2013;31:792-7.

21. Bisson A, Bodin A, Clementy N, Babuty D, Lip GYH and Fauchier L. Prediction of Incident Atrial Fibrillation According to Gender in Patients With Ischemic Stroke From a Nationwide Cohort. *Am J Cardiol*. 2018;121:437-444.

22. Choi YJ, Choi EK, Han KD, Jung JH, Park J, Lee E, Choe W, Lee SR, Cha MJ, Lim WH and Oh S. Temporal trends of the prevalence and incidence of atrial fibrillation and stroke among Asian patients with hypertrophic cardiomyopathy: A nationwide population-based study. *International Journal of Cardiology*. 2018;273:130-5.

23. Chyou JY, Hunter TD, Mollenkopf SA, Turakhia MP and Reynolds MR. Individual and Combined Risk Factors for Incident Atrial Fibrillation and Incident Stroke: An Analysis of 3 Million At-Risk US Patients. *J Am Heart Assoc*. 2015;4:e001723.

24. Cleland JG, Chattopadhyay S, Khand A, Houghton T and Kaye GC. Prevalence and incidence of arrhythmias and sudden death in heart failure. *Heart Fail Rev*. 2002;7:229-42.

25. Dharma Rao V, Rajaneesh Reddy M, Srikanth K, Raj Kumar Prakash B, Satya Prasad A and Guru Prasad SS. To study the prevalence and clinical profile of chronic atrial fibrillation in hospitalized patients. *Nitte University Journal of Health Science*. 2014;4:17-20.

26. Frontera A, Carpenter A, Ahmed N, Fasiolo M, Nelson M, Diab I, Cripps T, Thomas G and Duncan E. Demographic and Clinical Characteristics to Predict Paroxysmal Atrial Fibrillation: Insights from an Implantable Loop Recorder Population. *Pacing Clin Electrophysiol*. 2015;38:1217-22.

27. Goto S, Bhatt DL, Rother J, Alberts M, Hill MD, Ikeda Y, Uchiyama S, D'Agostino R, Ohman EM, Liau CS, Hirsch AT, Mas JL, Wilson PW, Corbalan R, Aichner F and Steg PG. Prevalence, clinical profile, and cardiovascular outcomes of atrial fibrillation patients with atherothrombosis. *Am Heart J*. 2008;156:855-63.

28. Healey JS, Martin JL, Duncan A, Connolly SJ, Ha AH, Morillo CA, Nair GM, Eikelboom J, Divakaramenon S and Dokainish H. Pacemaker-Detected Atrial Fibrillation in Patients With Pacemakers: Prevalence, Predictors, and Current Use of Oral Anticoagulation. *Canadian Journal of Cardiology*. 2013;29:224-228.

29. Horstmann S, Rizos T, Guntner J, Hug A, Jenetzky E, Krumsdorf U and Veltkamp R. Does the STAF score help detect paroxysmal atrial fibrillation in acute stroke patients? *Eur J Neurol*. 2013;20:147-52.

30. Jin Shyan W, Bau R, Ahmad F, Ismail R and Yean Yip AF. Prevalence of asymptomatic atrial fibrillation in Malaysian patients with hypertension. *Medical Journal of Malaysia*. 2013;68:141-143.

31. Jurkko R, Palojoki E, Huttunen H, Holm C, Lehto M, Helio T, Swan H and Toivonen L. Characteristics of atrial fibrillation and comorbidities in familial atrial fibrillation. *J Cardiovasc Electrophysiol*. 2013;24:768-74.

32. Khachab H and Brembilla-Perrot B. Prevalence of atrial fibrillation in patients with history of paroxysmal supraventricular tachycardia. *International Journal of Cardiology*. 2013;166:221-224.

33. Koton S and Rothwell PM. Performance of the ABCD and ABCD2 scores in TIA patients with carotid stenosis and atrial fibrillation. *Cerebrovasc Dis*. 2007;24:231-5.

34. Larstorp AC, Ariansen I, Gjesdal K, Olsen MH, Ibsen H, Devereux RB, Okin PM, Dahlof B, Kjeldsen SE and Wachtell K. Association of pulse pressure with new-onset atrial fibrillation in patients with hypertension and left ventricular hypertrophy: the Losartan Intervention For Endpoint (LIFE) reduction in hypertension study. *Hypertension*. 2012;60:347-53.

35. Tavernier R, Wolf M, Kataria V, Phlips T, Huys R, Taghji P, Louw R, Hoeyweghen RV, Vandekerckhove Y, Knecht S and Duytschaever M. Screening for atrial fibrillation in hospitalised geriatric patients. *Heart*. 2018;104:588-593.

36. Vinter N, Christesen AMS, Mortensen LS, Urbonaviciene G, Lindholt J, Johnsen SP and Frost L. Coronary artery calcium score and the long-term risk of atrial fibrillation in patients undergoing non-contrast cardiac computed tomography for suspected coronary artery disease: a Danish registry-based cohort study. *Eur Heart J Cardiovasc Imaging*. 2018;19:926-932.

37. Wakula P, Neumann B, Kienemund J, Thon-Gutschi E, Stojakovic T, Manninger M, Scherr D, Scharnagl H, Kapl M, Pieske B and Heinzel FR. CHA2DS2-VASc score and blood biomarkers to identify patients with atrial high-rate episodes and paroxysmal atrial fibrillation. *Europace*. 2017;19:544-551.

38. Zuo ML, Liu S, Chan KH, Lau KK, Chong BH, Lam KF, Chan YH, Lau YF, Lip GY, Lau CP, Tse HF and Siu CW. The CHADS2 and CHA 2DS 2-VASc scores predict new occurrence of atrial fibrillation and ischemic stroke. *J Interv Card Electrophysiol*. 2013;37:47-54.

39. Barrett TW, Storrow AB, Jenkins CA, Abraham RL, Liu D, Miller KF, Moser KM, Russ S, Roden DM, Harrell FE, Jr. and Darbar D. The AFFORD clinical decision aid to identify emergency department patients with atrial fibrillation at low risk for 30-day adverse events. *Am J Cardiol*. 2015;115:763-70.

40. Alonso A and Norby FL. Predicting Atrial Fibrillation and Its Complications. *Circ J*. 2016;80:1061-6.

41. Chan NY. Systematic screening for atrial fibrillation in the community: Evidence and obstacles. *Arrhythmia and Electrophysiology Review*. 2018;7:39-42.

42. Nguyen T, Waits G and Soliman EZ. The Role of Resting Electrocardiogram in Screening for Primary Prevention of Cardiovascular Diseases in High-Risk Groups. *Current Cardiovascular Risk Reports*. 2018;12:1-9.

43. Schnabel RB. Can we predict the occurrence of atrial fibrillation? *Clin Cardiol*. 2012;35 Suppl 1:5-9.

44. Yang Y, Zhang Z, Ng CY, Li G and Liu T. Meta-analysis of CHADS2 Score in Predicting Atrial Fibrillation. *Am J Cardiol*. 2015;116:554-62.

45. Ahmad Y, Lip GYH and Lane DA. Recent developments in understanding epidemiology and risk determinants of atrial fibrillation as a cause of stroke. *Canadian Journal of Cardiology*. 2013;29:S4-S13.

46. Allan V, Honarbakhsh S, Casas JP, Wallace J, Hunter R, Schilling R, Perel P, Morley K, Banerjee A and Hemingway H. Are cardiovascular risk factors also associated with the incidence of atrial fibrillation?: A systematic review and field synopsis of 23 factors in 32 population-based cohorts of 20 million participants. *Thrombosis and Haemostasis*. 2017;117:837-850.

47. Brugts JJ, Akin S, Helming AM, Loonstra S, Van Den Bos EJ and Kofflard MJM. The predictive value of cardiac biomarkers in prognosis and risk stratification of patients with atrial fibrillation. *Current Opinion in Cardiology*. 2011;26:449-456.

48. Dzeshka MS, Lane DA and Lip GY. Stroke and bleeding risk in atrial fibrillation: navigating the alphabet soup of risk-score acronyms (CHADS2 , CHA2 DS2 -VASc, R2 CHADS2 , HAS-BLED, ATRIA, and more). *Clin Cardiol*. 2014;37:634-44.

49. Gillis AM. Atrial Fibrillation and Ventricular Arrhythmias: Sex Differences in Electrophysiology, Epidemiology, Clinical Presentation, and Clinical Outcomes. *Circulation*. 2017;135:593-608.

50. Guenancia C, Garnier F, Mouhat B, Bejot Y, Maillot N, Fichot M, Fauchier L and Cottin Y. Screening and clinical implications of silent atrial fibrillation. *Revue de Medecine Interne*. 2018;39:574-579.

51. Jonas DE, Kahwati LC, Yun JDY, Cook Middleton J, Coker-Schwimmer M and Asher GN. Screening for atrial fibrillation with electrocardiography: Evidence report and systematic review for the US preventive services task force. *JAMA*. 2018;320:485-498.

52. Kaba RA, Camm AJ, Williams TM and Sharma R. Managing atrial fibrillation in the global community: The European perspective. *Global Cardiology Science and Practice*. 2013;24:173-84.

53. Kannel WB, Wolf PA, Benjamin EJ and Levy D. Prevalence, incidence, prognosis, and predisposing conditions for atrial fibrillation: population-based estimates. *Am J Cardiol*. 1998;82:2N-9N.

54. Lardizabal JA and Deedwania PC. Primary Prevention of Atrial Fibrillation - The Path Untread. *J Atr Fibrillation*. 2013;5:131-7.

55. Li Y, Pastori D, Guo Y, Wang Y and Lip GYH. Risk factors for new-onset atrial fibrillation: A focus on Asian populations. *International Journal of Cardiology*. 2018;261:92-98.

56. Mohlenkamp S, Wieneke H, Sack S and Erbel R. [Risk stratification of asymptomatic subjects using resting ECG and stress ECG]. *Herz*. 2007;32:362-70.

57. Moran PS, Flattery MJ, Teljeur C, Ryan M and Smith SM. Effectiveness of systematic screening for the detection of atrial fibrillation. *Cochrane Database of Systematic Reviews*. 2013;2013:CD009586.

58. Moran PS, Teljeur C, Ryan M and Smith SM. Systematic screening for the detection of atrial fibrillation. *Cochrane Database of Systematic Reviews*. 2016;6.

59. Rienstra M, McManus DD and Benjamin EJ. Novel risk factors for atrial fibrillation: useful for risk prediction and clinical decision making? *Circulation*. 2012;125:e941-6.

60. Sandhu RK and Healey JS. Screening for undiagnosed atrial fibrillation. *Expert Review of Cardiovascular Therapy*. 2018;16:591-598.

61. Valderrama AL, Dunbar SB and Mensah GA. Atrial fibrillation: Public health implications. *American Journal of Preventive Medicine*. 2005;29:75-80.

62. Vroomen M and Pison L. Lone atrial fibrillation: Risk factors, triggers and ablation techniques. *Journal of Atrial Fibrillation*. 2015;8:18-27.

63. Welton NJ, McAleenan A, Thom HH, Davies P, Hollingworth W, Higgins JP, Okoli G, Sterne JA, Feder G, Eaton D, Hingorani A, Fawsitt C, Lobban T, Bryden P, Richards A and Sofat R. Screening strategies for atrial fibrillation: a systematic review and cost-effectiveness analysis. *Health Technol Assess*. 2017;21:1-236.

64. Chang KCM, Lee JT, Vamos EP, Soljak M, Johnston D, Khunti K, Majeed A and Millett C. Impact of the National Health Service Health Check on cardiovascular disease risk: A difference-in-differences matching analysis. *Cmaj*. 2016;188:E228-E238.

65. Claes N, Van Laethem C, Goethals M, Goethals P, Mairesse G, Schwagten B, Nuyens D, Schrooten W and Vijgen J. Prevalence of atrial fibrillation in adults participating in a large-scale voluntary screening programme in Belgium. *Acta Cardiol*. 2012;67:273-8.

66. Dahl M, Frost L, Sogaard R, Klausen IC, Lorentzen V and Lindholt J. A population-based screening study for cardiovascular diseases and diabetes in Danish postmenopausal women: Acceptability and prevalence. *BMC Cardiovascular Disorders*. 2018;18:20.

67. Diederichsen ACP, Rasmussen LM, Sogaard R, Lambrechtsen J, Steffensen FH, Frost L, Egstrup K, Urbonaviciene G, Busk M, Olsen MH, Mickley H, Hallas J and Lindholt JS. The Danish Cardiovascular Screening Trial (DANCAVAS): Study protocol for a randomized controlled trial. *Trials*. 2015;16:554.

68. Hobbs FDR, Fitzmaurice DA, Mant J, Murray E, Jowett S, Bryan S, Raftery J, Davies M and Lip G. A randomised controlled trial and cost-effectiveness study of systematic screening (targeted and total population screening) versus routine practice for the detection of atrial fibrillation in people aged 65 and over. The SAFE study. *Health Technology Assessment*. 2005;9:iii-71.

69. Kaasenbrood F, Hollander M, Rutten FH, Gerhards LJ, Hoes AW and Tieleman RG. Yield of screening for atrial fibrillation in primary care with a hand-held, single-lead electrocardiogram device during influenza vaccination. *Europace*. 2016;18:1514-1520.

70. Lowres N, Neubeck L, Salkeld G, Krass I, McLachlan AJ, Redfern J, Bennett AA, Briffa T, Bauman A, Martinez C, Wallenhorst C, Lau JK, Brieger DB, Sy RW and Ben Freedman S. Feasibility and cost-effectiveness of stroke prevention through community screening for atrial fibrillation using iPhone ECG in pharmacies: The SEARCH-AF study. *Thrombosis and Haemostasis*. 2014;111:1167-1176.

71. Monteiro P. The SAFIRA study: A reflection on the prevalence and treatment patterns of atrial fibrillation and cardiovascular risk factors in 7500 elderly subjects. *Revista Portuguesa de Cardiologia*. 2018;37:307-313.

72. Morgan S and Mant D. Randomised trial of two approaches to screening for atrial fibrillation in UK general practice. *British Journal of General Practice*. 2002;52:373-374, 377-380.

73. Perula-de-Torres LA, Martinez-Adell MA, Gonzalez-Blanco V, Baena-Diez JM, Martin-Rioboo E, Parras-Rejano JM, Gonzalez-Lama J, Martin-Alvarez R, Ruiz-Moral R, Fernandez-Garcia JA, Perez-Diaz M, Ruiz-de-Castroviejo J, Perula-de-Torres C, Valero-Martin A, Roldan-Villalobos A, Criado-Larumbe M, Burdoy-Joaquin E, Coma-Sole M, Cervera-Leon M and Cuixart-Costa L. Opportunistic detection of atrial fibrillation in subjects aged 65 years or older in primare care: a randomised clinical trial of efficacy. DOFA-AP study protocol. *BMC Fam Pract*. 2012;13:106.

74. Proietti M, Mairesse GH, Goethals P, Scavee C, Vijgen J, Blankoff I, Vandekerckhove Y and Lip GYH. A population screening programme for atrial fibrillation: A report from the Belgian Heart Rhythm Week screening programme. *Europace*. 2016;18:1779-1786.

75. Smyth B, Marsden P, Corcoran R, Walsh R, Brennan C, McSharry K, Clarke J, Kelly PJ and Harbison J. Opportunistic screening for atrial fibrillation in a rural area. *QJM*. 2016;109:539-543.

76. Swancutt D, Hobbs R, Fitzmaurice D, Mant J, Murray E, Jowett S, Raftery J, Bryan S, Davies M and Lip G. A randomised controlled trial and cost effectiveness study of systematic screening (targeted and total population screening) versus routine practice for the detection of atrial fibrillation in the over 65s: (SAFE) [ISRCTNI9633732]. *BMC Cardiovascular Disorders*. 2004;4:12.

77. Turakhia MP, Ullal AJ, Hoang DD, Than CT, Miller JD, Friday KJ, Perez MV, Freeman JV, Wang PJ and Heidenreich PA. Feasibility of extended ambulatory electrocardiogram monitoring to identify silent atrial fibrillation in high-risk patients: the Screening Study for Undiagnosed Atrial Fibrillation (STUDY-AF). *Clin Cardiol*. 2015;38:285-92.

78. Veale EL, Stewart AJ, Mathie A, Lall SK, Rees-Roberts M, Savickas V, Bhamra SK and Corlett SA. Pharmacists detecting atrial fibrillation (PDAF) in primary care during the influenza vaccination season: A multisite, cross-sectional screening protocol. *BMJ Open*. 2018;8:e021121.

79. Virtanen R, Kryssi V, Vasankari T, Salminen M, Kivela SL and Airaksinen KEJ. Self-detection of atrial fibrillation in an aged population: The LietoAF Study. *European Journal of Preventive Cardiology*. 2014;21:1437-1442.

80. Sudlow M, Thomson R, Thwaites B, Rodgers H and Kenny RA. Prevalence of atrial fibrillation and eligibility for anticoagulants in the community. *Lancet*. 1998;352:1167-1171.

81. Aronsson M, Svennberg E, Rosenqvist M, Engdahl J, Al-Khalili F, Friberg L, Frykman V and Levin LA. Designing an optimal screening program for unknown atrial fibrillation: a cost-effectiveness analysis. *Europace*. 2017;19:1650-1656.

82. Aronsson M, Svennberg E, Rosenqvist M, Engdahl J, Al-Khalili F, Friberg L, Frykman-Kull V and Levin LA. Cost-effectiveness of mass screening for untreated atrial fibrillation using intermittent ECG recording. *Europace*. 2015;17:1023-9.

83. Jacobs MS, Kaasenbrood F, Postma MJ, van Hulst M and Tieleman RG. Cost-effectiveness of screening for atrial fibrillation in primary care with a handheld, single-lead electrocardiogram device in the Netherlands. *Europace*. 2018;20:12-18.

84. Maeda K, Shimbo T and Fukui T. Cost-effectiveness of a community-based screening programme for chronic atrial fibrillation in Japan. *J Med Screen*. 2004;11:97-102.

85. Moran PS, Teljeur C, Harrington P, Smith SM, Smyth B, Harbison J, Normand C and Ryan M. Cost-Effectiveness of a National Opportunistic Screening Program for Atrial Fibrillation in Ireland. *Value Health*. 2016;19:985-995.

86. Tarride JE, Dolovich L, Blackhouse G, Guertin JR, Burke N, Manja V, Grinvalds A, Lim T, Healey JS and Sandhu RK. Screening for atrial fibrillation in Canadian pharmacies: an economic evaluation. *CMAJ Open*. 2017;5:E653-E661.

87. Wu EQ, Birnbaum HG, Mareva M, Tuttle E, Castor AR, Jackman W and Ruskin J. Economic burden and co-morbidities of atrial fibrillation in a privately insured population. *Current Medical Research and Opinion*. 2005;21:1693-1699.

88. Andersson P, Londahl M, Abdon NJ and Terent A. The prevalence of atrial fibrillation in a geographically well-defined population in Northern Sweden: Implications for anticoagulation prophylaxis. *Journal of Internal Medicine*. 2012;272:170-176.

89. Aronow WS. Prevalence of heart disease in older women in a nursing home. *Journal of Women's Health*. 1998;7:1105-1112.

90. Aronow WS, Ahn C and Gutstein H. Prevalence of atrial fibrillation and association of atrial fibrillation with prior and new thromboembolic stroke in older patients. *Journal of the American Geriatrics Society*. 1996;44:521-523.

91. Ball J, Thompson DR, Ski CF, Carrington MJ, Gerber T and Stewart S. Estimating the current and future prevalence of atrial fibrillation in the Australian adult population. *Medical Journal of Australia*. 2015;202:32-36.

92. Berti D, Moors E, Moons P and Heidbuchel H. Prevalence and antithrombotic management of atrial fibrillation in hospitalised patients. *Heart (British Cardiac Society)*. 2015;101:884-893.

93. Bilato C, Corti MC, Baggio G, Rampazzo D, Cutolo A, Iliceto S and Crepaldi G. Prevalence, Functional Impact, and Mortality of Atrial Fibrillation in an Older Italian Population (from the Pro.V.A. Study). *American Journal of Cardiology*. 2009;104:1092-1097.

94. DeWilde S, Carey IM, Emmas C, Richards N and Cook DG. Trends in the prevalence of diagnosed atrial fibrillation, its treatment with anticoagulation and predictors of such treatment in UK primary care. *Heart*. 2006;92:1064-1070.

95. Friberg L and Bergfeldt L. Atrial fibrillation prevalence revisited. *Journal of Internal Medicine*. 2013;274:461-468.

96. Frishman WH, Heiman M, Karpenos A, Ooi WL, Mitzner A, Goldkorn R and Greenberg S. Twenty-four-hour ambulatory electrocardiography in elderly subjects: prevalence of various arrhythmias and prognostic implications (report from the Bronx Longitudinal Aging Study). *Am Heart J*. 1996;132:297-302.

97. Go AS, Hylek EM, Phillips KA, Chang YC, Henault LE, Selby JV and Singer DE. Prevalence of diagnosed atrial fibrillation in adults: National implications for rhythm management and stroke prevention: The anticoagulation and risk factors in atrial fibrillation (ATRIA) study. *Journal of the American Medical Association*. 2001;285:2370-2375.

98. Khan MA, Raja S, Ibrahim MS and Hammersely C. Prevalence and management of atrial fibrillation in primary care: a case study. *Prim Health Care Res Dev*. 2014;15:355-61.

99. Lee SR, Choi EK, Han KD, Cha MJ and Oh S. Trends in the incidence and prevalence of atrial fibrillation and estimated thromboembolic risk using the CHA<inf>2</inf>DS<inf>2</inf>-VASc score in the entire Korean population. *International Journal of Cardiology*. 2017;236:226-231.

100. Miyasaka Y, Barnes ME, Gersh BJ, Cha SS, Bailey KR, Abhayaratna WP, Seward JB and Tsang TS. Secular trends in incidence of atrial fibrillation in Olmsted County, Minnesota, 1980 to 2000, and implications on the projections for future prevalence. *Circulation*. 2006;114:119-25.

101. Muthalaly RG, Koplan BA, Albano A, North C, Campbell JI, Kakuhikire B, Vorechovska D, Kraemer JD, Tsai AC and Siedner MJ. Low population prevalence of atrial fibrillation in rural Uganda: A community-based cross-sectional study. *International Journal of Cardiology*. 2018;271:87-91.

102. Nakamura S, Adachi H, Enomoto M, Fukami A, Kumagai E, Nohara Y, Kono S, Nakao E, Sakaue A, Tsuru T, Morikawa N and Fukumoto Y. Trends in coronary risk factors and electrocardiogram findings from 1977 to 2009 with 10-year mortality in Japanese elderly males - The Tanushimaru Study. *Journal of Cardiology*. 2017;70:353-358.

103. Norberg J, Backstrom S, Jansson J and Johansson L. Estimating the prevalence of atrial fibrillation in a general population using validated electronic health data. *Clinical Epidemiology*. 2013;5:475-481.

104. Reardon G, Nelson WW, Patel AA, Philpot T and Neidecker M. Prevalence of Atrial Fibrillation in US Nursing Homes: Results from the National Nursing Home Survey, 1985-2004. *Journal of the American Medical Directors Association*. 2012;13:529-534.

105. Turakhia MP, Shafrin J, Bognar K, Trocio J, Abdulsattar Y, Wiederkehr D and Goldman DP. Estimated prevalence of undiagnosed atrial fibrillation in the United States. *PLoS ONE*. 2018;13:e0195088.

106. Vermond RA, Geelhoed B, Verweij N, Tieleman RG, Van der Harst P, Hillege HL, Van Gilst WH, Van Gelder IC and Rienstra M. Incidence of Atrial Fibrillation and Relationship With Cardiovascular Events, Heart Failure, and Mortality: A Community-Based Study From the Netherlands. *J Am Coll Cardiol*. 2015;66:1000-7.

107. Wheeldon NM, Tayler DI, Anagnostou E, Cook D, Wales C and Oakley GDG. Screening for atrial fibrillation in primary care. *Heart*. 1998;79:50-55.

108. Williams BA, Honushefsky AM and Berger PB. Temporal Trends in the Incidence, Prevalence, and Survival of Patients With Atrial Fibrillation From 2004 to 2016. *Am J Cardiol*. 2017;120:1961-1965.

109. Yang PS, Ryu S, Kim D, Jang E, Yu HT, Kim TH, Hwang J, Joung B and Lip GYH. Variations of Prevalence and Incidence of Atrial Fibrillation and Oral Anticoagulation Rate According to Different Analysis Approaches. *Sci Rep*. 2018;8:6856.

110. Adamsson Eryd S, Ostling G, Rosvall M, Persson M, Smith JG, Melander O, Hedblad B and Engstrom G. Carotid intima-media thickness is associated with incidence of hospitalized atrial fibrillation. *Atherosclerosis*. 2014;233:673-8.

111. Alonso A, Agarwal SK, Soliman EZ, Ambrose M, Chamberlain AM, Prineas RJ and Folsom AR. Incidence of atrial fibrillation in whites and African-Americans: The Atherosclerosis Risk in Communities (ARIC) study. *American Heart Journal*. 2009;158:111-117.

112. Alonso A, Misialek JR, Eckfeldt JH, Selvin E, Coresh J, Chen LY, Soliman EZ, Agarwal SK and Lutsey PL. Circulating fibroblast growth factor-23 and the incidence of atrial fibrillation: the Atherosclerosis Risk in Communities study. *Journal of the American Heart Association*. 2014;3:e001082.

113. Aronis KN, Zhao D, Hoogeveen RC, Alonso A, Ballantyne CM, Guallar E, Jones SR, Martin SS, Nazarian S, Steffen BT, Virani SS and Michos ED. Associations of Lipoprotein(a) Levels With Incident Atrial Fibrillation and Ischemic Stroke: The ARIC (Atherosclerosis Risk in Communities) Study. *J Am Heart Assoc*. 2017;6:e007372.

114. Benjamin EJ, Levy D, Vaziri SM, D'Agostino RB, Belanger AJ and Wolf PA. Independent risk factors for atrial fibrillation in a population-based cohort. The Framingham Heart Study. *Jama*. 1994;271:840-4.

115. Brembilla-Perrot B, Laporte F, Sellal JM, Schwartz J, Olivier A, Zinzius PY, Manenti V, Beurrier D, Andronache M, Louis P, Selton O, De La Chaise AT and De Chillou C. 1:1 atrial-flutter. Prevalence and clinical characteristics. *International Journal of Cardiology*. 2013;168:3287-3290.

116. Cea-Calvo L, Redon J, Lozano JV, Fernandez-Perez C, Marti-Canales JC, Llisterri JL, Gonzalez-Esteban J and Aznar J. [Prevalence of atrial fibrillation in the Spanish population aged 60 years or more. The PREV-ICTUS study]. *Rev Esp Cardiol*. 2007;60:616-24.

117. Chamberlain AM, Gersh BJ, Alonso A, Chen LY, Berardi C, Manemann SM, Killian JM, Weston SA and Roger VL. Decade-long trends in atrial fibrillation incidence and survival: A community study. *American Journal of Medicine*. 2015;128:260-267.

118. Chan NY and Choy CC. Screening for atrial fibrillation in 13 122 Hong Kong citizens with smartphone electrocardiogram. *Heart*. 2017;103:24-31.

119. Chei CL, Raman P, Ching CK, Yin ZX, Shi XM, Zeng Y and Matchar DB. Prevalence and risk factors of atrial fibrillation in chinese elderly: Results from the chinese longitudinal healthy longevity survey. *Chinese Medical Journal*. 2015;128:2426-2432.

120. Davis RC, Hobbs FDR, Kenkre JE, Roalfe AK, Iles R, Lip GYH and Davies MK. Prevalence of atrial fibrillation in the general population and in high-risk groups: The ECHOES study. *Europace*. 2012;14:1553-1559.

121. Diouf I, Magliano DJ, Carrington MJ, Stewart S and Shaw JE. Prevalence, incidence, risk factors and treatment of atrial fibrillation in Australia: The Australian Diabetes, Obesity and Lifestyle (AusDiab) longitudinal, population cohort study. *Int J Cardiol*. 2016;205:127-132.

122. Disertori M, Lombardi F, Barlera S, Latini R, Maggioni AP, Zeni P, Di Pasquale G, Cosmi F and Franzosi MG. Clinical predictors of atrial fibrillation recurrence in the Gruppo Italiano per lo Studio della Sopravvivenza nell'Infarto Miocardico-Atrial Fibrillation (GISSI-AF) trial. *Am Heart J*. 2010;159:857-63.

123. Filippi A, Bettoncelli G and Zaninelli A. Detected atrial fibrillation in North Italy: Rates, calculated stroke risk and proportion of patients receiving thrombo-prophylaxis. *Family Practice*. 2000;17:337-339.

124. Friberg J, Scharling H, Gadsboll N and Jensen GB. Sex-specific increase in the prevalence of atrial fibrillation (The Copenhagen City Heart Study). *American Journal of Cardiology*. 2003;92:1419-1423.

125. Gill PS, Calvert M, Davis R, Davies MK, Freemantle N and Lip GYH. Prevalence of heart failure and atrial fibrillation in minority ethnic subjects: The Ethnic-Echocardiographic heart of England screening study (E-ECHOES). *PLoS ONE*. 2011;6:e26710.

126. Gillott RG, Willan K, Kain K, Sivananthan UM and Tayebjee MH. South Asian ethnicity is associated with a lower prevalence of atrial fibrillation despite greater prevalence of established risk factors: A population-based study in Bradford Metropolitan District. *Europace*. 2017;19:356-363.

127. Guize L, Thomas F, Bean K, Benetos A and Pannier B. [Atrial fibrillation: prevalence, risk factors and mortality in a large French population with 15 years of follow-up]. *Bull Acad Natl Med*. 2007;191:791-803.

128. Kim HJ, Young KO, Sung J, Kim JH, Song YB, Lee WS, Choi JO, Shin DH, Cho SW, Choi JH, Hahn JY and Kim JS. Risk factors for predicting new-onset atrial fibrillation in persons who received health screening tests. *Korean Circulation Journal*. 2007;37:609-615.

129. Krahn AD, Manfreda J, Tate RB, Mathewson FA and Cuddy TE. The natural history of atrial fibrillation: incidence, risk factors, and prognosis in the Manitoba Follow-Up Study. *Am J Med*. 1995;98:476-84.

130. Kurl S, Ala-Kopsala M, Ruskoaho H, Makikallio T, Nyyssonen K, Vuolteenaho O, Sivenius J, Salonen JT and Laukkanen JA. Plasma N-terminal fragments of natriuretic peptides predict the risk of stroke and atrial fibrillation in men. *Heart*. 2009;95:1067-71.

131. Latini R, Staszewsky L, Sun JL, Bethel MA, Disertori M, Haffner SM, Holman RR, Chang F, Giles TD, Maggioni AP, Rutten GE, Standl E, Thomas L, Tognoni G, Califf RM and McMurray JJ. Incidence of atrial fibrillation in a population with impaired glucose tolerance: the contribution of glucose metabolism and other risk factors. A post hoc analysis of the Nateglinide and Valsartan in Impaired Glucose Tolerance Outcomes Research trial. *Am Heart J*. 2013;166:935-40.

132. Lau CP, Gbadebo TD, Connolly SJ, Van Gelder IC, Capucci A, Gold MR, Israel CW, Morillo CA, Siu CW, Abe H, Carlson M, Tse HF, Hohnloser SH and Healey JS. Ethnic differences in atrial fibrillation identified using implanted cardiac devices. *Journal of Cardiovascular Electrophysiology*. 2013;24:381-387.

133. Lehtonen AO, Langen VL, Puukka PJ, Kahonen M, Nieminen MS, Jula AM and Niiranen TJ. Incidence rates, correlates, and prognosis of electrocardiographic P-wave abnormalities - a nationwide population-based study. *J Electrocardiol*. 2017;50:925-932.

134. Li L, Mao H, Ishwaran H, Rajeswaran J, Ehrlinger J and Blackstone EH. Estimating the prevalence of atrial fibrillation from a three-class mixture model for repeated diagnoses. *Biom J*. 2017;59:331-343.

135. Li Y, Wu YF, Chen KP, Li X, Zhang X, Xie GQ, Wang FZ and Zhang S. Prevalence of atrial fibrillation in China and its risk factors. *Biomed Environ Sci*. 2013;26:709-16.

136. Lip GYH, Bawden L, Hodson R, Rutland E, Snatchfold J and Beevers DG. Atrial fibrillation amongst the Indo-Asian general practice population. The West Birmingham Atrial Fibrillation Project. *International Journal of Cardiology*. 1998;65:187-192.

137. Lip GYH, Golding DJ, Nazir M, Gareth Beevers D, Child DL and Fletcher RI. A survey of atrial fibrillation in general practice: The west Birmingham atrial fibrillation project. *British Journal of General Practice*. 1997;47:285-289.

138. MacFarlane PW, Murray H, Sattar N, Stott DJ, Ford I, Buckley B, Jukema JW, Westendorp RGJ and Shepherd J. The incidence and risk factors for new onset atrial fibrillation in the PROSPER study. *Europace*. 2011;13:634-639.

139. Mandalenakis Z, Eriksson H, Welin L, Caidahl K, Dellborg M, Rosengren A, Lappas G, Hedner J, Johansson S, Svardsudd K and Hansson PO. Atrial natriuretic peptide as a predictor of atrial fibrillation in a male population study. The Study of Men Born in 1913 and 1923. *Int J Cardiol*. 2014;171:44-8.

140. Mareedu RK, Abdalrahman IB, Dharmashankar KC, Granada JF, Chyou PH, Sharma PP, Smith PN, Hayes JJ, Greenlee RT and Vidaillet H. Atrial flutter versus atrial fibrillation in a general population: Differences in comorbidities associated with their respective onset. *Clinical Medicine and Research*. 2010;8:1-6.

141. Mullenix PS, Martin MJ, Steele SR, Lavenson Jr GS, Starnes BW, Hadro NC, Peterson RP and Andersen CA. Rapid high-volume population screening for three major risk factors of future stroke: Phase I results. *Vascular and Endovascular Surgery*. 2006;40:177-187.

142. Murphy NF, Simpson CR, Jhund PS, Stewart S, Kirkpatrick M, Chalmers J, MacIntyre K and McMurray JJ. A national survey of the prevalence, incidence, primary care burden and treatment of atrial fibrillation in Scotland. *Heart*. 2007;93:606-12.

143. Nichols GA, Reinier K and Chugh SS. Independent contribution of diabetes to increased prevalence and incidence of atrial fibrillation. *Diabetes Care*. 2009;32:1851-1856.

144. Okin PM, Wachtell K, Kjeldsen SE, Julius S, Lindholm LH, Dahlof B, Hille DA, Nieminen MS, Edelman JM and Devereux RB. Incidence of atrial fibrillation in relation to changing heart rate over time in hypertensive patients: the LIFE study. *Circ Arrhythm Electrophysiol*. 2008;1:337-43.

145. O'Neal WT, Judd SE, Limdi NA, McIntyre WF, Kleindorfer DO, Cushman M, Howard VJ, Howard G and Soliman EZ. Differential Impact of Risk Factors in Blacks and Whites in the Development of Atrial Fibrillation: the Reasons for Geographic And Racial Differences in Stroke (REGARDS) Study. *Journal of racial and ethnic health disparities*. 2017;4:718-724.

146. Patton KK, Ellinor PT, Heckbert SR, Christenson RH, DeFilippi C, Gottdiener JS and Kronmal RA. N-terminal pro-B-type natriuretic peptide is a major predictor of the development of atrial fibrillation: the Cardiovascular Health Study. *Circulation*. 2009;120:1768-74.

147. Patton KK, Heckbert SR, Alonso A, Bahrami H, Lima JA, Burke G and Kronmal RA. N-terminal pro-B-type natriuretic peptide as a predictor of incident atrial fibrillation in the Multi-Ethnic Study of Atherosclerosis: the effects of age, sex and ethnicity. *Heart*. 2013;99:1832-6.

148. Perez MV, Wang PJ, Larson JC, Soliman EZ, Limacher M, Rodriguez B, Klein L, Manson JE, Martin LW, Prineas R, Connelly S, Hlatky M, Wassertheil-Smoller S and Stefanick ML. Risk factors for atrial fibrillation and their population burden in postmenopausal women: The Women's Health Initiative Observational Study. *Heart*. 2013;99:1173-1178.

149. Peters SAE and Woodward M. Established and novel risk factors for atrial fibrillation in women compared with men. *Heart*. 2018;105:226-34.

150. Psaty BM, Manolio TA, Kuller LH, Kronmal RA, Cushman M, Fried LP, White R, Furberg CD and Rautaharju PM. Incidence of and risk factors for atrial fibrillation in older adults. *Circulation*. 1997;96:2455-2461.

151. Rohrbacker NJ, Kleinman NL, White SA, March JL and Reynolds MR. The burden of atrial fibrillation and other cardiac arrhythmias in an employed population: Associated costs, absences, and objective productivity loss. *Journal of Occupational and Environmental Medicine*. 2010;52:383-391.

152. Ruigomez A, Johansson S, Wallander MA and Rodriguez LAG. Incidence of chronic atrial fibrillation in general practice and its treatment pattern. *Journal of Clinical Epidemiology*. 2002;55:358-363.

153. Schnabel RB, Johannsen SS, Wild PS and Blankenberg S. [Prevalence and risk factors of atrial fibrillation in Germany : data from the Gutenberg Health Study]. *Herz*. 2015;40:8-15.

154. Schnabel RB, Larson MG, Yamamoto JF, Kathiresan S, Rong J, Levy D, Keaney JF, Jr., Wang TJ, Vasan RS and Benjamin EJ. Relation of multiple inflammatory biomarkers to incident atrial fibrillation. *Am J Cardiol*. 2009;104:92-6.

155. Schnabel RB, Yin X, Gona P, Larson MG, Beiser AS, McManus DD, Newton-Cheh C, Lubitz SA, Magnani JW, Ellinor PT, Seshadri S, Wolf PA, Vasan RS, Benjamin EJ and Levy D. 50 year trends in atrial fibrillation prevalence, incidence, risk factors, and mortality in the Framingham Heart Study: a cohort study. *Lancet*. 2015;386:154-62.

156. Shulman E, Kargoli F, Aagaard P, Hoch E, Di Biase L, Fisher J, Gross J, Kim S, Ferrick KJ and Krumerman A. Socioeconomic status and the development of atrial fibrillation in Hispanics, African Americans and non-Hispanic whites. *Clinical Cardiology*. 2017;40:770-776.

157. Son MK, Lim NK, Cho MC and Park HY. Incidence and Risk Factors for Atrial Fibrillation in Korea: the National Health Insurance Service Database (2002-2010). *Korean Circ J*. 2016;46:515-21.

158. Stewart S, Hart CL, Hole DJ and McMurray JJV. Population prevalence, incidence, and predictors of atrial fibrillation in the Renfrew/Paisley study. *Heart*. 2001;86:516-521.

159. Sun GZ, Guo L, Wang XZ, Song HJ, Li Z, Wang J and Sun YX. Prevalence of atrial fibrillation and its risk factors in rural China: a cross-sectional study. *International journal of cardiology*. 2015;182:13-17.

160. Takase H, Dohi Y, Sonoda H and Kimura G. Prediction of atrial fibrillation by B-type natriuretic peptide. *Journal of Atrial Fibrillation*. 2013;5:44-52.

161. Tamariz L, Agarwal S, Soliman EZ, Chamberlain AM, Prineas R, Folsom AR, Ambrose M and Alonso A. Association of serum uric acid with incident atrial fibrillation (from the Atherosclerosis Risk in Communities [ARIC] study). *American Journal of Cardiology*. 2011;108:1272-1276.

162. Thacker EL, McKnight B, Psaty BM, Longstreth Jr WT, Dublin S, Jensen PN, Newton KM, Smith NL, Siscovick DS and Heckbert SR. Association of body mass index, diabetes, hypertension, and blood pressure levels with risk of permanent atrial fibrillation. *Journal of General Internal Medicine*. 2013;28:247-253.

163. Yang Y, Han X, Chen Y, Gao L, Yin X, Li H, Qiu J, Wang Y, Zhou Y and Xia Y. Association between modifiable lifestyle and the prevalence of atrial fibrillation in a Chinese population: Based on the cardiovascular health score. *Clinical Cardiology*. 2017;40:1061-1067.

164. Zhou Z and Hu D. An epidemiological study on the prevalence of atrial fibrillation in the Chinese population of mainland China. *Journal of Epidemiology*. 2008;18:209-216.

165. de Ruijter W, Westendorp RG, Macfarlane PW, Jukema JW, Assendelft WJ and Gussekloo J. The routine electrocardiogram for cardiovascular risk stratification in old age: the Leiden 85-plus study. *J Am Geriatr Soc*. 2007;55:872-7.

166. O'Neal WT, Efird JT, Judd SE, McClure LA, Howard VJ, Howard G and Soliman EZ. Impact of Awareness and Patterns of Nonhospitalized Atrial Fibrillation on the Risk of Mortality: The Reasons for Geographic And Racial Differences in Stroke (REGARDS) Study. *Clin Cardiol*. 2016;39:103-10.

167. Aizawa Y, Watanabe H and Okumura K. Electrocardiogram (ECG) for the Prediction of Incident Atrial Fibrillation: An Overview. *J Atr Fibrillation*. 2017;10:1724.

168. Bury G, Swan D, Cullen W, Keane D, Tobin H, Egan M, Fitzmaurice D, Carberry C and Kelleher C. Screening for atrial fibrillation in general practice: A national, cross-sectional study of an innovative technology. *International Journal of Cardiology*. 2015;178:247-252.

169. Busch MC, Gross S, Alte D, Kors JA, Volzke H, Ittermann T, Werner A, Kruger A, Busch R, Dorr M and Felix SB. Impact of atrial fibrillation detected by extended monitoring-A population-based cohort study. *Annals of Noninvasive Electrocardiology*. 2017;22 e12453.

170. Chan NY, Choy CC, Chan CK and Siu CW. Effectiveness of a nongovernmental organization-led large-scale community atrial fibrillation screening program using the smartphone electrocardiogram: An observational cohort study. *Heart Rhythm*. 2018;15:1306-1311.

171. Doliwa PS, Frykman V and Rosenqvist M. Short-term ECG for out of hospital detection of silent atrial fibrillation episodes. *Scandinavian Cardiovascular Journal*. 2009;43:163-168.

172. Engdahl J, Svennberg E, Friberg L, Al-Khalili F, Frykman V, Gudmundsdottir KK, Fredriksson T and Rosenqvist M. Stepwise mass screening for atrial fibrillation using N-terminal pro b-type natriuretic peptide: The STROKESTOP II study design. *Europace*. 2017;19:297-302.

173. Gonzalez Blanco V, Perula de Torres LA, Martin Rioboo E, Martinez Adell MA, Parras Rejano JM, Gonzalez Lama J, Ruiz Moruno J, Martin Alvarez R, Fernandez Garcia JA, Ruiz de Castroviejo J, Roldan Villalobos A and Ruiz Moral R. Opportunistic screening for atrial fibrillation versus detecting symptomatic patients aged 65 years and older: A cluster-controlled clinical trial. *Med Clin (Barc)*. 2017;148:8-15.

174. Granada J, Uribe W, Chyou PH, Maassen K, Vierkant R, Smith PN, Hayes J, Eaker E and Vidaillet H. Incidence and predictors of atrial flutter in the general population. *Journal of the American College of Cardiology*. 2000;36:2242-2246.

175. Haberman ZC, Jahn RT, Bose R, Tun H, Shinbane JS, Doshi RN, Chang PM and Saxon LA. Wireless smartphone ECG enables large-scale screening in diverse populations. *Journal of Cardiovascular Electrophysiology*. 2015;26:520-526.

176. Halcox JPJ, Wareham K, Cardew A, Gilmore M, Barry JP, Phillips C and Gravenor MB. Assessment of Remote Heart Rhythm Sampling Using the AliveCor Heart Monitor to Screen for Atrial Fibrillation: The REHEARSE-AF Study. *Circulation*. 2017;136:1784-1794.

177. Hald J, Poulsen PB, Qvist I, Holm L, Wedell-Wedellsborg D, Dybro L and Frost L. Opportunistic screening for atrial fibrillation in a real-life setting in general practice in Denmark - The Atrial Fibrillation Found On Routine Detection (AFFORD) non-interventional study. *PLoS ONE*. 2017;12:e0188086.

178. Jaakkola J, Vasankari T, Virtanen R and Juhani Airaksinen KE. Reliability of pulse palpation in the detection of atrial fibrillation in an elderly population. *Scandinavian journal of primary health care*. 2017;35:293-298.

179. Kearley K, Selwood M, Van den Bruel A, Thompson M, Mant D, Hobbs FR, Fitzmaurice D and Heneghan C. Triage tests for identifying atrial fibrillation in primary care: a diagnostic accuracy study comparing single-lead ECG and modified BP monitors. *BMJ Open*. 2014;4:e004565.

180. Lown M, Yue A, Lewith G, Little P and Moore M. Screening for Atrial Fibrillation using Economical and accurate TechnologY (SAFETY)-a pilot study. *BMJ Open*. 2017;7:e013535.

181. Mant J, Fitzmaurice DA, Hobbs FDR, Jowett S, Murray ET, Holder R, Davies M and Lip GYH. Accuracy of diagnosing atrial fibrillation on electrocardiogram by primary care practitioners and interpretative diagnostic software: Analysis of data from screening for atrial fibrillation in the elderly (SAFE) trial. *British Medical Journal*. 2007;335:380-382.

182. Mei B, Jiefu Y and Yingying L. Serum N-terminal-pro-brain natriuretic peptide level and its clinical implications in patients with atrial fibrillation. *Clinical Cardiology*. 2009;32:E1-E5.

183. Motorina SV and Kalinichenko AN. Real-time Algorithm for Detection of Atrial Fibrillation. *Biomedical Engineering*. 2016;50:161-165.

184. Munschauer FE, Hens MM, Priore RL, Stolarski E, Buffamonte S, Carlin A, Wechsler L, Massaro L, Barch C, Hughes R, Anderson A, Sung G, Baker S and Limon S. Screening for atrial fibrillation in the community: a multicenter validation trial. *J Stroke Cerebrovasc Dis*. 1999;8:99-103.

185. Nakamura M, Endo H, Nasu M, Arakawa N, Segawa T and Hiramori K. Value of plasma B type natriuretic peptide measurement for heart disease screening in a Japanese population. *Heart*. 2002;87:131-5.

186. Nakamura M, Tanaka F, Sato K, Segawa T and Nagano M. B-type natriuretic peptide testing for structural heart disease screening: a general population-based study. *J Card Fail*. 2005;11:705-12.

187. Omboni S and Verberk WJ. Opportunistic screening of atrial fibrillation by automatic blood pressure measurement in the community. *BMJ Open*. 2016;6:e010745.

188. Somerville S, Somerville J, Croft P and Lewis M. Atrial fibrillation: A comparison of methods to identify cases in general practice. *British Journal of General Practice*. 2000;50:727-729.

189. Steinhubl SR, Mehta RR, Ebner GS, Ballesteros MM, Waalen J, Steinberg G, Van Crocker P, Felicione E, Carter CT, Edmonds S, Honcz JP, Miralles GD, Talantov D, Sarich TC and Topol EJ. Rationale and design of a home-based trial using wearable sensors to detect asymptomatic atrial fibrillation in a targeted population: The mHealth Screening to Prevent Strokes (mSToPS) trial. *American Heart Journal*. 2016;175:77-85.

190. Taggar JS, Coleman T, Lewis S, Heneghan C and Jones M. Accuracy of methods for diagnosing atrial fibrillation using 12-lead ECG: A systematic review and meta-analysis. *International journal of cardiology*. 2015;184:175-183.

191. Taggar JS, Coleman T, Lewis S, Heneghan C and Jones M. Accuracy of methods for detecting an irregular pulse and suspected atrial fibrillation: A systematic review and meta-analysis. *European Journal of Preventive Cardiology*. 2016;23:1330-1338.

192. Tieleman RG, Plantinga Y, Rinkes D, Bartels GL, Posma JL, Cator R, Hofman C and Houben RP. Validation and clinical use of a novel diagnostic device for screening of atrial fibrillation. *Europace*. 2014;16:1291-1295.

193. Aakre CA, McLeod CJ, Cha SS, Tsang TS, Lip GY and Gersh BJ. Comparison of clinical risk stratification for predicting stroke and thromboembolism in atrial fibrillation. *Stroke*. 2014;45:426-31.

194. Apenteng PN, Murray ET, Holder R, Hobbs FDR and Fitzmaurice DA. An international longitudinal registry of patients with atrial fibrillation at risk of stroke (GARFIELD): The UK protocol. *BMC Cardiovascular Disorders*. 2013;13:1-8.

195. Aspberg S, Chang Y, Atterman A, Bottai M, Go AS and Singer DE. Comparison of the ATRIA, CHADS2, and CHA2DS2-VASc stroke risk scores in predicting ischaemic stroke in a large Swedish cohort of patients with atrial fibrillation. *Eur Heart J*. 2016;37:3203-3210.

196. Bodapati RK, Kizer JR, Kop WJ, Kamel H and Stein PK. Addition of 24-hour heart rate variability parameters to the cardiovascular health study stroke risk score and prediction of incident stroke: The cardiovascular health study. *Journal of the American Heart Association*. 2017;6:e004305.

197. Cha MJ, Cho Y, Oh IY, Choi EK and Oh S. Validation of Conventional Thromboembolic Risk Factors in a Korean Atrial Fibrillation Population- Suggestion for a Novel Scoring System, CHA2DS2-VAK. *Circ J*. 2018;82:2970-5.

198. Chen T, Yang YM, Tan HQ, Liang Y and Zhu J. Baseline characteristics and 1-year follow-up of chinese atrial fibrillation patients according to age: A registry study. *PACE - Pacing and Clinical Electrophysiology*. 2014;37:1392-1403.

199. Coppens M, Eikelboom JW, Hart RG, Yusuf S, Lip GY, Dorian P, Shestakovska O and Connolly SJ. The CHA2DS2-VASc score identifies those patients with atrial fibrillation and a CHADS2 score of 1 who are unlikely to benefit from oral anticoagulant therapy. *Eur Heart J*. 2013;34:170-6.

200. Fitzmaurice DA, McCahon D, Baker J, Murray ET, Jowett S, Sandhar H, Holder RL and Hobbs FDR. Is screening for AF worthwhile? Stroke risk in a screened population from the SAFE study. *Family Practice*. 2014;31:298-302.

201. Ghazal F, Theobald H, Rosenqvist M and Al-Khalili F. Feasibility and outcomes of atrial fibrillation screening using intermittent electrocardiography in a primary healthcare setting: A cross-sectional study. *PLoS One*. 2018;13:e0198069.

202. Groot A, Bots ML, Rutten FH, den Ruijter HM, Numans ME and Vaartjes I. Measurement of ECG abnormalities and cardiovascular risk classification: a cohort study of primary care patients in the Netherlands. *Br J Gen Pract*. 2015;65:e1-8.

203. Kim TH, Yang PS, Kim D, Yu HT, Uhm JS, Kim JY, Pak HN, Lee MH, Joung B and Lip GYH. CHA2DS2-VASc Score for Identifying Truly Low-Risk Atrial Fibrillation for Stroke: A Korean Nationwide Cohort Study. *Stroke*. 2017;48:2984-2990.

204. Min SN, Park SJ, Kim DJ, Subramaniyam M and Lee KS. Development of an Algorithm for Stroke Prediction: A National Health Insurance Database Study in Korea. *European Neurology*. 2018;79:214-220.

205. Nakamura M, Koeda Y, Tanaka F, Onoda T, Itai K, Ohsawa M, Tanno K, Sakata K, Omama S, Ishibashi Y, Makita S, Ohta M, Ogasawara K, Komatsu T and Okayama A. Plasma B-type natriuretic peptide as a predictor of cardiovascular events in subjects with atrial fibrillation: a community-based study. *PLoS One*. 2013;8:e81243.

206. Rietbrock S, Heeley E, Plumb J and van Staa T. Chronic atrial fibrillation: Incidence, prevalence, and prediction of stroke using the Congestive heart failure, Hypertension, Age >75, Diabetes mellitus, and prior Stroke or transient ischemic attack (CHADS2) risk stratification scheme. *American Heart Journal*. 2008;156:57-64.

207. Tomlin AM, Lloyd HS and Tilyard MW. Atrial fibrillation in New Zealand primary care: Prevalence, risk factors for stroke and the management of thromboembolic risk. *Eur J Prev Cardiol*. 2017;24:311-319.

208. Vanassche T, Lauw MN, Eikelboom JW, Healey JS, Hart RG, Alings M, Avezum A, Diaz R, Hohnloser SH, Lewis BS, Shestakovska O, Wang J and Connolly SJ. Risk of ischaemic stroke according to pattern of atrial fibrillation: analysis of 6563 aspirin-treated patients in ACTIVE-A and AVERROES. *Eur Heart J*. 2015;36:281-7.

209. AbouEzzeddine OF, McKie PM, Scott CG, Rodeheffer RJ, Chen HH, Michael Felker G, Jaffe AS, Burnett JC and Redfield MM. Biomarker-based risk prediction in the community. *Eur J Heart Fail*. 2016;18:1342-1350.

210. Park GM, Han S, Kim SH, Jo MW, Her SH, Lee JB, Lee MS, Kim HC, Ahn JM, Lee SW, Kim YH, Kim BJ, Koh JM, Kim HK, Choe J, Park SW and Park SJ. Model for assessing cardiovascular risk in a Korean population. *Circ Cardiovasc Qual Outcomes*. 2014;7:944-51.

211. Park GM and Kim YH. Model for predicting cardiovascular disease: Insights from a Korean cardiovascular risk model. *Pulse*. 2015;3:153-157.

212. Mehta S, Jackson R, Pylypchuk R, Poppe K, Wells S and Kerr AJ. Development and validation of alternative cardiovascular risk prediction equations for population health planning: a routine health data linkage study of 1.7 million New Zealanders. *Int J Epidemiol*. 2018;47:1571-84.

213. Cabrera S, Valles E, Benito B, Alcalde O, Jimenez J, Fan R and Marti-Almor J. Simple predictors for new onset atrial fibrillation. *Int J Cardiol*. 2016;221:515-20.

214. Langley P, Dewhurst M, Di Marco LY, Adams P, Dewhurst F, Mwita JC, Walker R and Murray A. Accuracy of algorithms for detection of atrial fibrillation from short duration beat interval recordings. *Med Eng Phys*. 2012;34:1441-7.

215. Nasir JM, Pomeroy W, Marler A, Hann M, Baykaner T, Jones R, Stoll R, Hursey K, Meadows A, Walker J and Kindsvater S. Predicting Determinants of Atrial Fibrillation or Flutter for Therapy Elucidation in Patients at Risk for Thromboembolic Events (PREDATE AF) Study. *Heart Rhythm*. 2017;14:955-961.

216. Tu K, Nieuwlaat R, Cheng SY, Wing L, Ivers N, Atzema CL, Healey JS and Dorian P. Identifying Patients With Atrial Fibrillation in Administrative Data. *Can J Cardiol*. 2016;32:1561-1565.

217. Alves-Cabratosa L, Garcia-Gil M, Comas-Cufi M, Ponjoan A, Marti R, Parramon D, Blanch J and Ramos R. Incident Atrial Fibrillation Hazard in Hypertensive Population: A Risk Function from and for Clinical Practice. *Hypertension*. 2015;65:1180-1186.

218. Alonso A, Roetker NS, Soliman EZ, Chen LY, Greenland P and Heckbert SR. Prediction of Atrial Fibrillation in a Racially Diverse Cohort: The Multi-Ethnic Study of Atherosclerosis (MESA). *J Am Heart Assoc*. 2016;5:e003077.

219. Chao TF, Liu CJ, Chen SJ, Wang KL, Lin YJ, Chang SL, Lo LW, Hu YF, Tuan TC, Wu TJ, Chen TJ and Chen SA. CHADS2 score and risk of new-onset atrial fibrillation: a nationwide cohort study in Taiwan. *Int J Cardiol*. 2013;168:1360-3.

220. Christophersen IE, Yin X, Larson MG, Lubitz SA, Magnani JW, McManus DD, Ellinor PT and Benjamin EJ. A comparison of the CHARGE-AF and the CHA2DS2-VASc risk scores for prediction of atrial fibrillation in the Framingham Heart Study. *Am Heart J*. 2016;178:45-54.

221. Garg PK, O'Neal WT, Chen LY, Loehr LR, Sotoodehnia N, Soliman EZ and Alonso A. American Heart Association's Life Simple 7 and Risk of Atrial Fibrillation in a Population Without Known Cardiovascular Disease: The ARIC (Atherosclerosis Risk in Communities) Study. *J Am Heart Assoc*. 2018;7:e008424.

222. Guo Y, Tian Y, Wang H, Si Q, Wang Y and Lip GYH. Prevalence, incidence, and lifetime risk of atrial fibrillation in China: New insights into the global burden of atrial fibrillation. *Chest*. 2015;147:109-119.

223. Kolek MJ, Graves AJ, Xu M, Bian A, Teixeira PL, Shoemaker MB, Parvez B, Xu H, Heckbert SR, Ellinor PT, Benjamin EJ, Alonso A, Denny JC, Moons KGM, Shintani AK, Harrell FE, Roden DM and Darbar D. Evaluation of a prediction model for the development of atrial fibrillation in a repository of electronic medical records. *JAMA Cardiology*. 2016;1:1007-1013.

224. Lubitz SA, Yin X, Fontes JD, Magnani JW, Rienstra M, Pai M, Villalon ML, Vasan RS, Pencina MJ, Levy D, Larson MG, Ellinor PT and Benjamin EJ. Association between familial atrial fibrillation and risk of new-onset atrial fibrillation. *Jama*. 2010;304:2263-9.

225. Maheshwari A, Norby FL, Soliman EZ, Koene R, Rooney M, O'Neal WT, Alonso A and Chen LY. Refining Prediction of Atrial Fibrillation Risk in the General Population With Analysis of P-Wave Axis (from the Atherosclerosis Risk in Communities Study). *Am J Cardiol*. 2017;120:1980-1984.

226. Pfister R, Bragelmann J, Michels G, Wareham NJ, Luben R and Khaw KT. Performance of the CHARGE-AF risk model for incident atrial fibrillation in the EPIC Norfolk cohort. *Eur J Prev Cardiol*. 2015;22:932-9.

227. Saliba W, Gronich N, Barnett-Griness O and Rennert G. Usefulness of CHADS2 and CHA2DS2-VASc Scores in the Prediction of New-Onset Atrial Fibrillation: A Population-Based Study. *Am J Med*. 2016;129:843-9.

228. Schnabel RB, Aspelund T, Li G, Sullivan LM, Suchy-Dicey A, Harris TB, Pencina MJ, D'Agostino RB, Sr., Levy D, Kannel WB, Wang TJ, Kronmal RA, Wolf PA, Burke GL, Launer LJ, Vasan RS, Psaty BM, Benjamin EJ, Gudnason V and Heckbert SR. Validation of an atrial fibrillation risk algorithm in whites and African Americans. *Arch Intern Med*. 2010;170:1909-17.

229. Shulman E, Kargoli F, Aagaard P, Hoch E, Di Biase L, Fisher J, Gross J, Kim S, Krumerman A and Ferrick KJ. Validation of the Framingham Heart Study and CHARGE-AF Risk Scores for Atrial Fibrillation in Hispanics, African-Americans, and Non-Hispanic Whites. *Am J Cardiol*. 2016;117:76-83.

230. Suenari K, Chao TF, Liu CJ, Kihara Y, Chen TJ and Chen SA. Usefulness of HATCH score in the prediction of new-onset atrial fibrillation for Asians. *Medicine*. 2017;96:e5597.

231. Svennberg E, Lindahl B, Berglund L, Eggers KM, Venge P, Zethelius B, Rosenqvist M, Lind L and Hijazi Z. NT-proBNP is a powerful predictor for incident atrial fibrillation - Validation of a multimarker approach. *Int J Cardiol*. 2016;223:74-81.

232. Tischer TS, Schneider R, Lauschke J, Diedrich D, Kundt G and Bansch D. Prevalence of atrial fibrillation and the HATCH score: Intensified monitoring of patients with high HATCH score. *Herz*. 2015;40:803-808.

233. Tischer TS, Schneider R, Lauschke J, Nesselmann C, Klemm A, Diedrich D, Kundt G and Bansch D. Prevalence of atrial fibrillation in patients with high CHADS2- and CHA2DS2VASc-scores: anticoagulate or monitor high-risk patients? *Pacing Clin Electrophysiol*. 2014;37:1651-7.

234. Berntsson J, Smith JG, Nilsson PM, Hedblad B, Melander O and Engstrom G. Pro-atrial natriuretic peptide and prediction of atrial fibrillation and stroke: The Malmo Preventive Project. *Eur J Prev Cardiol*. 2017;24:788-795.

235. Di Benedetto L, Michels G, Luben R, Khaw KT and Pfister R. Individual and combined impact of lifestyle factors on atrial fibrillation in apparently healthy men and women: The EPIC-Norfolk prospective population study. *European Journal of Preventive Cardiology*. 2018;25:1374-1383.

236. Filion KB, Agarwal SK, Ballantyne CM, Eberg M, Hoogeveen RC, Huxley RR, Loehr LR, Nambi V, Soliman EZ and Alonso A. High-sensitivity cardiac troponin T and the risk of incident atrial fibrillation: the Atherosclerosis Risk in Communities (ARIC) study. *Am Heart J*. 2015;169:31-8.

237. Li L, Selvin E, Lutsey PL, Hoogeveen RC, O'Neal WT, Soliman EZ, Chen LY and Alonso A. Association of N-terminal pro B-type natriuretic peptide (NT-proBNP) change with the risk of atrial fibrillation in the ARIC cohort. *Am Heart J*. 2018;204:119-127.

238. O'Neal WT, Efird JT, Dawood FZ, Yeboah J, Alonso A, Heckbert SR and Soliman EZ. Coronary artery calcium and risk of atrial fibrillation (from the multi-ethnic study of atherosclerosis). *Am J Cardiol*. 2014;114:1707-12.

239. O'Neal WT, Efird JT, Nazarian S, Alonso A, Heckbert SR and Soliman EZ. Mitral annular calcification and incident atrial fibrillation in the Multi-Ethnic Study of Atherosclerosis. *Europace*. 2015;17:358-63.

240. Rahman F, Yin X, Larson MG, Ellinor PT, Lubitz SA, Vasan RS, McManus DD, Magnani JW and Benjamin EJ. Trajectories of Risk Factors and Risk of New-Onset Atrial Fibrillation in the Framingham Heart Study. *Hypertension*. 2016;68:597-605.

241. Rienstra M, Geelhoed B, Yin X, Siland JE, Vermond RA, Mulder BA, Van Der Harst P, Hillege HL, Benjamin EJ and Van Gelder IC. Cluster Individuals Based on Phenotype and Determine the Risk for Atrial Fibrillation in the PREVEND and Framingham Heart Study Populations. *PLoS One*. 2016;11:e0165828.

242. Rienstra M, Yin X, Larson MG, Fontes JD, Magnani JW, McManus DD, McCabe EL, Coglianese EE, Amponsah M, Ho JE, Januzzi JL, Jr., Wollert KC, Fradley MG, Vasan RS, Ellinor PT, Wang TJ and Benjamin EJ. Relation between soluble ST2, growth differentiation factor-15, and high-sensitivity troponin I and incident atrial fibrillation. *Am Heart J*. 2014;167:109-115.

243. Schnabel RB, Wild PS, Wilde S, Ojeda FM, Schulz A, Zeller T, Sinning CR, Kunde J, Lackner KJ, Munzel T and Blankenberg S. Multiple biomarkers and atrial fibrillation in the general population. *PLoS One*. 2014;9:e112486.

244. Sinner MF, Stepas KA, Moser CB, Krijthe BP, Aspelund T, Sotoodehnia N, Fontes JD, Janssens AC, Kronmal RA, Magnani JW, Witteman JC, Chamberlain AM, Lubitz SA, Schnabel RB, Vasan RS, Wang TJ, Agarwal SK, McManus DD, Franco OH, Yin X, Larson MG, Burke GL, Launer LJ, Hofman A, Levy D, Gottdiener JS, Kaab S, Couper D, Harris TB, Astor BC, Ballantyne CM, Hoogeveen RC, Arai AE, Soliman EZ, Ellinor PT, Stricker BH, Gudnason V, Heckbert SR, Pencina MJ, Benjamin EJ and Alonso A. B-type natriuretic peptide and C-reactive protein in the prediction of atrial fibrillation risk: the CHARGE-AF Consortium of community-based cohort studies. *Europace*. 2014;16:1426-33.

245. Sinner MF, Wang N, Fox CS, Fontes JD, Rienstra M, Magnani JW, Vasan RS, Calderwood AH, Pencina M, Sullivan LM, Ellinor PT and Benjamin EJ. Relation of circulating liver transaminase concentrations to risk of new-onset atrial fibrillation. *American Journal of Cardiology*. 2013;111:219-224.

246. Smith JG, Newton-Cheh C, Almgren P, Struck J, Morgenthaler NG, Bergmann A, Platonov PG, Hedblad B, Engstrom G, Wang TJ and Melander O. Assessment of conventional cardiovascular risk factors and multiple biomarkers for the prediction of incident heart failure and atrial fibrillation. *J Am Coll Cardiol*. 2010;56:1712-9.

247. Muse ED, Wineinger NE, Spencer EG, Peters M, Henderson R, Zhang Y, Barrett PM, Rivera SP, Wohlgemuth JG, Devlin JJ, Shiffman D and Topol EJ. Validation of a genetic risk score for atrial fibrillation: A prospective multicenter cohort study. *PLoS Medicine*. 2018;15:e1002525.

248. Tada H, Shiffman D, Smith JG, Sjogren M, Lubitz SA, Ellinor PT, Louie JZ, Catanese JJ, Engstrom G, Devlin JJ, Kathiresan S and Melander O. Twelve-single nucleotide polymorphism genetic risk score identifies individuals at increased risk for future atrial fibrillation and stroke. *Stroke*. 2014;45:2856-2862.

249. Schnabel RB, Larson MG, Yamamoto JF, Sullivan LM, Pencina MJ, Meigs JB, Tofler GH, Selhub J, Jacques PF, Wolf PA, Magnani JW, Ellinor PT, Wang TJ, Levy D, Vasan RS and Benjamin EJ. Relations of biomarkers of distinct pathophysiological pathways and atrial fibrillation incidence in the community. *Circulation*. 2010;121:200-7.

1. Search #4 was adapted from: Geersing GJ, Bouwmeester W, Zuithoff P, Spijker R, Leeflang M, Moons KG. Search filters for finding prognostic and diagnostic prediction studies in Medline to enhance systematic reviews. PLoS One. 2012;7(2):e32844. doi: 10.1371/journal.pone.0032844. PMID: 22393453). [↑](#footnote-ref-1)
2. https://www.nice.org.uk/guidance/ng50/documents/search-strategies [↑](#footnote-ref-2)
